# An integrative approach unveils FOSL1 as an oncogene vulnerability in KRAS-driven lung and pancreatic cancer

**DOI:** 10.1038/ncomms14294

**Published:** 2017-02-21

**Authors:** Adrian Vallejo, Naiara Perurena, Elisabet Guruceaga, Pawel K. Mazur, Susana Martinez-Canarias, Carolina Zandueta, Karmele Valencia, Andrea Arricibita, Dana Gwinn, Leanne C. Sayles, Chen-Hua Chuang, Laura Guembe, Peter Bailey, David K. Chang, Andrew Biankin, Mariano Ponz-Sarvise, Jesper B. Andersen, Purvesh Khatri, Aline Bozec, E. Alejandro Sweet-Cordero, Julien Sage, Fernando Lecanda, Silve Vicent

**Affiliations:** 1 University of Navarra, Center for Applied Medical Research, Program in Solid Tumors and Biomarkers, Pamplona 31010, Spain; 2 University of Navarra, Center for Applied Medical Research, Proteomics, Genomics and Bioinformatics Core Facility, Pamplona 31010, Spain; 3 Department of Genetics, Stanford University School of Medicine, Stanford, California 94305, USA; 4 Department of Pediatrics, Stanford University School of Medicine, Stanford, California 94305, USA; 5 Department of Pathology, Stanford University School of Medicine, Stanford, California 94305, USA; 6 University of Navarra, Center for Applied Medical Research, Morphology Unit, Pamplona 31010, Spain; 7 Wolfson Wohl Cancer Research Centre, Institute of Cancer Sciences, University of Glasgow, Garscube Estate, Switchback Road, Bearsden, Glasgow G61 1BD, UK; 8 West of Scotland Pancreatic Unit, Glasgow Royal Infirmary, Glasgow G31 2ER, UK; 9 The Kinghorn Cancer Centre, Cancer Division, Garvan Institute of Medical Research, University of New South Wales, 384 Victoria St, Darlinghurst, Sydney, New South Wales 2010, Australia; 10 Department of Surgery, Bankstown Hospital, Eldridge Road, Bankstown, Sydney, New South Wales 2200, Australia; 11 South Western Sydney Clinical School, Faculty of Medicine, University of New South Wales, Liverpool, New South Wales 2170, Australia; 12 Clínica Universidad de Navarra, Department of Medical Oncology, Pamplona 31008, Spain; 13 Biotech Research and Innovation Center, University of Copenhagen, Copenhagen, DK-2200, Denmark; 14 Stanford Institute for Immunity, Transplantation and Infection, Stanford, California 94305, USA; 15 Stanford Center for Biomedical Informatics Research, Department of Medicine, Stanford University, Stanford, California 94305, USA; 16 Department of Internal Medicine 3 and Institute of Clinical Immunology, University of Erlangen-Nuremberg, 91054 Erlangen, Germany; 17 IdiSNA, Navarra Institute for Health Research, Pamplona 31008, Spain; 18 University of Navarra, Department of Histology and Pathology, Pamplona 31008, Spain

## Abstract

KRAS mutated tumours represent a large fraction of human cancers, but the vast majority remains refractory to current clinical therapies. Thus, a deeper understanding of the molecular mechanisms triggered by KRAS oncogene may yield alternative therapeutic strategies. Here we report the identification of a common transcriptional signature across mutant KRAS cancers of distinct tissue origin that includes the transcription factor FOSL1. High FOSL1 expression identifies mutant KRAS lung and pancreatic cancer patients with the worst survival outcome. Furthermore, FOSL1 genetic inhibition is detrimental to both KRAS-driven tumour types. Mechanistically, FOSL1 links the KRAS oncogene to components of the mitotic machinery, a pathway previously postulated to function orthogonally to oncogenic KRAS. FOSL1 targets include AURKA, whose inhibition impairs viability of mutant KRAS cells. Lastly, combination of AURKA and MEK inhibitors induces a deleterious effect on mutant KRAS cells. Our findings unveil KRAS downstream effectors that provide opportunities to treat KRAS-driven cancers.

## Abstract (web summary)

KRAS-driven cancers remain refractory to current clinical therapies. In this study, the authors show that lung and pancreatic cancers expressing oncogenic KRAS can be targeted by genetic inhibition of FOSL1, which involves downregulation of genes of the mitotic machinery.

K*RAS* is frequently mutated in human cancers^1^ and is a driver of neoplasias in the lung, pancreas, colon and biliary tract^2^,^3^,^4^,^5^, where tumour maintenance is highly dependent on continued oncogenic KRAS expression^6^,^7^. Unfortunately, recent approaches to inhibit KRAS directly still lack long-term inhibition capacity and show toxicity *in vivo*^8^. Thus alternative strategies to neutralize KRAS-mediated effects are urgently needed. Design of such strategies would be facilitated by a deeper understanding of the molecular alterations downstream of oncogenic KRAS.

Gene-expression profiling provides a tractable strategy to expose novel components of the oncogenic KRAS network. KRAS signatures from single experimental systems or tumour types that classify patients according to KRAS genotype^9^,^10^ and KRAS dependency^11^ have been described, but show little overlap^10^. This suggests that the transcriptional response is highly dependent on the tissue of origin and raises the question whether a core of genes relevant for mutant KRAS biology is preserved across different tumour types. Integration of multiple laboratory and clinical gene-expression data from different tissues spanning mouse and human species would address this question but has yet not been described.

A plethora of transcription factors have been linked to transformation by KRAS oncogene *in vitro*, including the AP1 transcription complex^12^,^13^,^14^. Conversely, little evidence exists for a critical activity of AP1 or other transcription factors in KRAS-driven tumorigenesis. In this regard, loss of WT1, FOXM1 or MYC has been reported to impair initiation of mutant *Kras* lung or pancreatic tumours in mice^15^,^16^,^17^,^18^. Nonetheless, given the complexity of the transcriptional regulatory network elicited by KRAS oncogene, it is likely that other transcription factors could play a role in KRAS-driven tumorigenesis and represent novel vulnerabilities.

Human tumours expressing KRAS oncogene undergo mitotic stress^19^ and, thus, are highly dependent on the mitotic machinery to preserve cellular homeostasis. In this cellular context, various components of the mitotic machinery have been unveiled as KRAS vulnerabilities in human cancer^20^,^21^. However, whether KRAS oncogene intersects with the mitosis network influencing the cellular adaptation to mitotic stress is largely unknown. Therefore, the mitotic machinery has been postulated to operate orthogonally to mutant KRAS signalling^20^,^22^.

In this study, we devised an integrative strategy to expose common core elements of KRAS signalling critical for homeostasis of *KRAS* mutated tumours by combining (1) a cross-tumours gene-expression screen to identify KRAS-dependent candidate genes, and (2) patient outcome to inform selection of candidate genes for functional follow-up. This strategy unveiled FOSL1 as a prominent component of oncogenic KRAS-driven lung and pancreatic ductal adenocarcinoma (PDAC), which partially functions by transcriptionally regulating a subset of genes involved in mitotic fitness that unveils opportunities for pharmacological intervention.

## Results

### Identification of candidate genes by a cross-tumours screen

**Figure 1:**
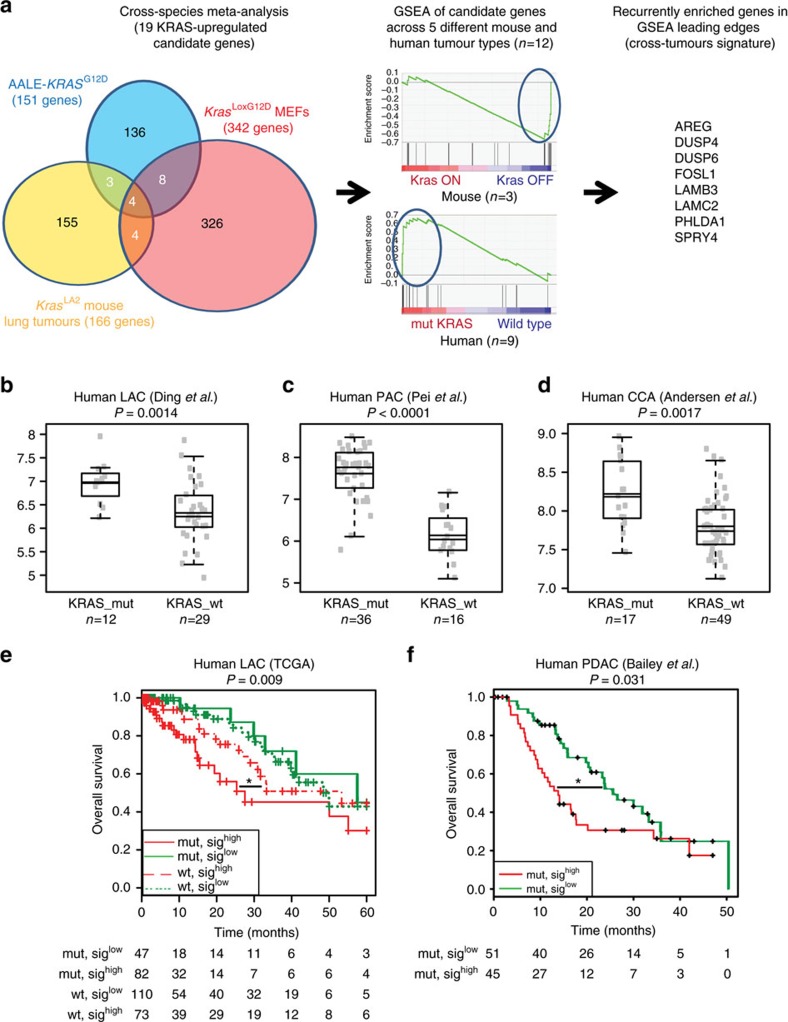
Identification of cross-tumours KRAS-dependent genes. (**a**) Workflow of the gene-expression strategy for the identification of mutant *KRAS*-regulated genes. (Left) Venn's diagram summarizing the cross-species meta-analysis. In white are genes over-expressed in mutant *KRAS* cells or tumours over wild-type *KRAS* controls (*n*=19). Blue: Human lung immortalized bronchoepithelial cells (AALE cells) expressing exogenous *KRAS*^G12D^ compared to wild-type *KRAS*-expressing cells. Orange: *Kras*^LA2^ mouse LAC tumours compared to normal lung tissue. Red: *Kras*-activated mouse embryo fibroblasts compared to wild-type *Kras* mouse embryo fibroblasts. (Center) GSEA graphs showing the number of mouse and human cancer data sets used to query the relevance of the initial 19 candidate genes identified. (Right) An eight-gene cross-tumours KRAS signature including genes recurrently found in more than 50% of the GSEA leading edges. (**b**–**d**) Box plots of classification analyses based on the expression of the eight-gene cross-tumours signature in LAC, PDAC and CCA data sets. *P* values obtained using Student's *t*-test. (**e**) Kaplan–Meier plot of LAC patients for the expression of the eight-gene cross-tumours signature taking into account *KRAS* status. Wild-type group excluded patients with genetic alterations in non-KRAS oncogene drivers to prevent any bias in survival due to administration of targeted therapies. *P* values obtained using the log-rank test (Mantel Cox). (**f**) Kaplan–Meier plot of PDAC patients for the expression of the eight-gene cross-tumours signature. *P* values obtained using the log-rank test (Mantel-Cox). *P<0.05.

To uncover a core of genes consistently regulated by KRAS across mouse and human tumours, we followed a two-tiered approach. First, we screened gene-expression data from *in vitro* and *in vivo* experimental systems of epithelial and mesenchymal origin with either wild-type or mutant *KRAS* allele (human immortalized bronchoepithelial cells, *Kras*^LSLG12D/+^ mouse embryo fibroblasts and a mouse model of *Kras*-driven lung cancer). Only 19 genes were consistently overexpressed in mutant *KRAS* phenotype in at least two of the three studies (Fig. 1a, Supplementary Fig. 1a and Supplementary Table 1), indicating that the transcriptional response to oncogenic *KRAS* is markedly influenced by the background of each experimental system. Also, only three genes (*DUSP6*, *GLRX* and *PHLDA1*) overlapped with a previous cross-species KRAS signature^23^, underscoring the validity of our approach to uncover novel KRAS-regulated genes.

Next, Gene Set Enrichment Analysis (GSEA) of publicly available data from mouse and human tumours was performed to investigate the relevance of the 19 candidate genes in cancer (Fig. 1a). A negative (−) enrichment was observed in mouse lung adenocarcinoma (LAC) and PDAC where mutant *KRAS* expression was depleted^2^,^7^ (Supplementary Fig. 1b,c). In human cancer, a positive (+) enrichment was observed in mutant *KRAS* patients compared to wild-type in LAC (*n*=3), PDAC, colorectal cancer (CRC), cholangiocarcinoma (CCA) and multiple myeloma (MM) (Supplementary Fig. 1d–j). These observations were recapitulated in human LAC and CRC cell lines from the Cancer Cell Line Encyclopedia^24^ (Supplementary Fig. 1k,l, and Supplementary Table 2). The results suggest that the 19 genes are regulated through a cell-autonomous mechanism and are representative of KRAS-driven cancers independently of the tissue origin.

### A cross-tumours signature predicts KRAS status and survival

To refine the value of the identified KRAS-regulated genes to human cancer, those genes recurrently present in >50% of the leading edges of the mouse and human cancer GSEA were identified (Supplementary Table 2). This analysis across different KRAS-driven tumours unveiled a core of eight genes including *AREG*, *DUSP4*, *DUSP6*, *FOSL1*, *LAMB3*, *LAMC2*, *PHLDA1* and *SPRY4*. Notably, *LAMB3* has been reported as a synthetic lethal interaction with oncogenic *KRAS* in colon cancer^21^, suggesting that other co-identified genes could also display a functional role in mutant *KRAS* cancer.

Next, we interrogated independent data sets of KRAS-driven cancers to determine whether the eight-gene signature would be a predictor for *KRAS* status. A higher geometric mean was representative of LAC (*n*=3), PDAC and CCA tumours harbouring *KRAS* mutations (Fig. 1b–d and Supplementary Fig. 1m). The results correlated with a statistically significant area under receiver operating characteristic (AUC) (Supplementary Fig. 1n). Since *KRAS* mutations are mutually exclusive with other dominant oncogenic drivers in LAC, we reasoned that the cross-tumours eight-gene signature would be exclusive of the *KRAS* phenotype. The geometric mean of the eight genes did not discriminate mutant *EGFR* from wild-type patients in four data sets (Supplementary Fig. 1o). Similar results were observed for *AML4-ALK*, *BRAF* and *DDR2* mutant or *MYC* amplified tumours in The Cancer Genome Atlas (TCGA) data set (Supplementary Fig. 1p).

We then sought to investigate the role of the eight-gene signature in patient survival using the TCGA LAC data set that is large enough to stratify patients based on *KRAS* status. Mutant *KRAS* patients expressing high levels of the eight-gene signature had the worst outcome (Fig. 1e) with a significant decreased survival compared to wild-type patients (*P*=0.04, log-rank test, Mantel Cox). Moreover, multivariate analysis of mutant *KRAS* patients expressing high levels of the eight-gene signature showed that the effect of gene expression on patient survival is irrespective of stage, age and gender (*P*=0.003, HR=1.599 (1.177–2.172), Cox proportional hazards model), conversely to what was found in wild-type *KRAS* patients (*P*=0.883, HR=1.024 (0.748–1.402), Cox proportional hazards model). The eight-gene signature was also associated with poor survival in PDAC patients^25^ (Fig. 1f), whereas no association with patient survival was observed in other tumours where KRAS mutations are rarely found or absent such as squamous cell lung carcinoma or breast cancer (Supplementary Fig. 1q,r). These results argue that high expression of the eight-gene signature is representative of mutant *KRAS* cancers.

### FOSL1 upregulation in KRAS-driven human LAC

**Figure 2:**
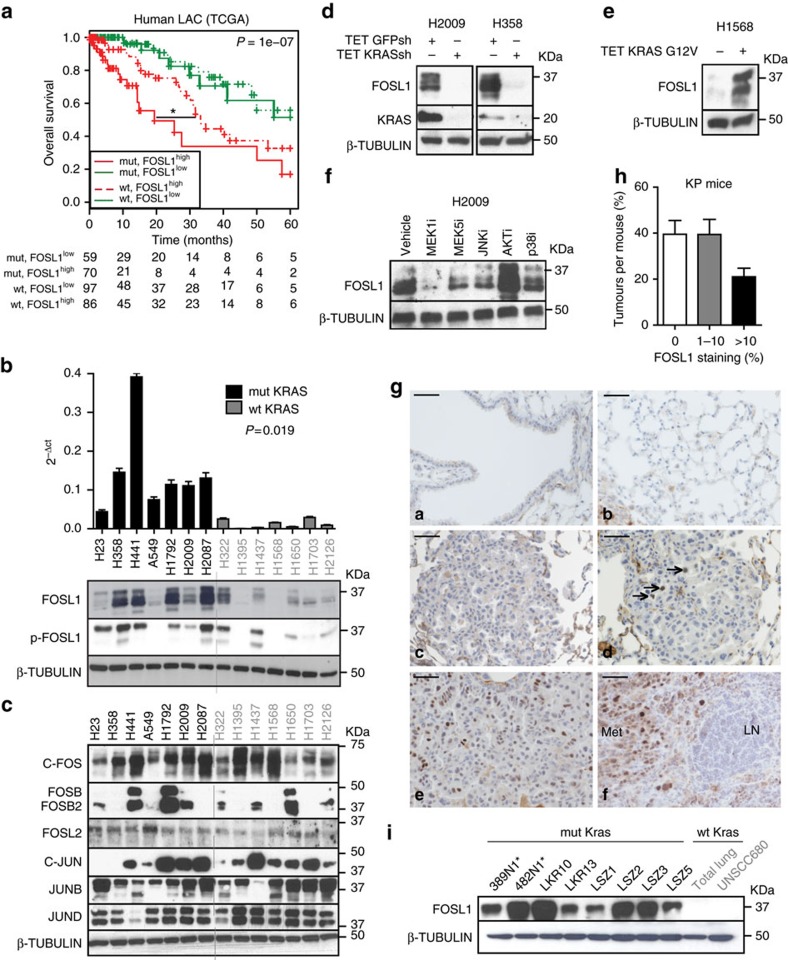
Upregulation of FOSL1 in LAC. (**a**) Kaplan–Meier plot of the LAC TCGA data set for FOSL1 expression based on *KRAS* status. Wild-type group excluded patients with genetic alterations in non-KRAS oncogene drivers to prevent outcome bias due to patients treated with targeted therapies. *P* values obtained using the log-rank test (Mantel-Cox). *P<0.05. (**b**) mRNA and protein analyses by qPCR and western blot, respectively, for indicated genes and proteins in a panel of mutant (*n*=7) and wild-type (*n*=7) *KRAS* cell lines. mRNA levels were compared by Student's *t*-test. (**c**) Western blot analysis for members of the FOS and JUN families. (**d**) Western blot showing FOSL1 expression levels after KRAS inhibition with a specific shRNA in mutant *KRAS* cells (H2009 and H358). (**e**) Western blot to detect FOSL1 protein expression upon KRAS expression in wild-type KRAS cells (H1568). (**f**) Western blot of H2009 cells treated with U0126 (MEKi, 10 μM), BIX02189 (MEK5i, 10 μM), SB203580 (JNKi, 20 μM), LY294002 (AKTi, 10 μM) and SB203580 (p38i, 20 μM) and probed with indicated antibodies. (**g**) Expression of FOSL1 by immunohistochemistry in *Kras*^LSLG12D^*, p53*^f/f^ mice (*n*=5; a: normal alveolar epithelium; b: normal bronchiolar epithelium; c,d: adenoma; e: adenocarcinoma; f: lymph node tumour metastasis (Met)). Scale bar 50 μm. (**h**) Quantification of FOSL1 staining in lung tumours of *Kras*^LSLG12D^*, p53*^f/f^(KP) mice. (**i**) FOSL1 protein expression in mouse LAC cells derived from *Kras*^LA2^ (LKR10, LKR13), *Kras*^LSLG12D^ (LSZ1-5) and *Kras*^*LSLG12D*^*, p53*^f/f^ (389N1, 482N1), squamous cell lung carcinoma cells (UNSCC680) and normal lung. Asterisk indicates metastatic cell lines isolated from the lymph nodes. QPCR plots and western blot images are representative of three independent experiments with different cell lysates. Error bars correspond to s.d.

To further delineate the contribution of the eight candidate genes, we carried out single gene survival analysis to discriminate those genes involved in the clinical outcome of mutant *KRAS* LAC patients. We focused on LAC where patients can be stratified based on KRAS genotype. Only high FOSL1 expression identified a group of patients bearing *KRAS* mutations with the worst survival outcome and the differences between wild-type and mutant *KRAS* patients expressing high *FOSL1* levels were statistically significant (*P*=0.016, log-rank test, Mantel Cox) (Fig. 2a). Multivariate analysis including stage, age and sex showed that *FOSL1* expression was an independent survival marker in LAC patients with *KRAS* mutations (*P*=0.006; HR=5.072 (1.61–15.98), Cox proportional hazards model). No association was observed between FOSL1 expression and survival of squamous lung carcinoma or breast cancer patients (Supplementary Fig. 2a,b). These results suggest that FOSL1 expression is preferentially associated with *KRAS* mutations.

*FOSL1* (also known as FOS-related antigen 1, *FRA-1*) is a transcription factor that heterodimerizes with members of the JUN family to form the AP1 transcription complex^26^, which is upregulated during KRAS-induced transformation of murine fibroblasts *in vitro*^27^,^28^. However, its role in human KRAS-driven cancer remains unclear. To investigate *FOSL1* in human LAC, a panel of cell lines with known *RAS* status was utilized. *FOSL1* mRNA and protein were upregulated in mutant *RAS* cell lines compared to wild type (Fig. 2b). Likewise, phosphorylation of FOSL1, previously suggested to be a mechanism of FOSL1 activation^19^, was preferentially observed in mutant cells. Of note, analysis of other AP1 members of the JUN and FOS families revealed no obvious genotype-specific expression (Fig. 2c). Further analysis of patient-derived xenografts revealed upregulation of FOSL1 expression in tumours with *KRAS* mutations compared to wild type (Supplementary Fig. 2c).

To explore whether FOSL1 was downstream of mutant *KRAS*, KRAS inhibition was carried out in different cell lines. KRAS knockdown lowered FOSL1 protein levels (Fig. 2d). Conversely, FOSL1 was upregulated upon mutant *KRAS* overexpression in wild-type KRAS cells (Fig. 2e). ERK1/2 has been reported as the main KRAS effector controlling FOSL1 expression in KRAS-driven cancer^29^. Pharmacological inhibition of KRAS canonical effectors showed that, in addition to ERK1/2, ERK5 and JNK consistently regulate FOSL1 expression in mutant *KRAS* cell lines (Fig. 2f and Supplementary Fig. 2d). Thus, FOSL1 is regulated by, and integrates signals from, different kinases downstream of KRAS.

### FOSL1 overexpression in a model of LAC

To study FOSL1 expression in a more physiologically relevant system, a genetically engineered mouse model of *Kras*-driven LAC, *Kras*^LSL-G12D^; *Trp53*^f/f^ (KP), that is representative of human disease was used. Immunohistochemistry analysis was performed in normal and tumour tissue after antibody validation (Supplementary Fig. 2e). No FOSL1 expression was detected in normal alveolar and bronchiolar epithelia of KP mice (Fig. 2g). Conversely, around 60% of lung tumours expressed FOSL1 to some extent (Fig. 2h). Of note, FOSL1 expression was also found in advanced tumour lesions such as lymph node metastases (*n*=4) (Fig. 2g). Interestingly, only a small fraction of cells expressing FOSL1 was positive for Ki67 (7.25%) (Supplementary Fig. 2f), suggesting that FOSL1 does not specifically label a population of proliferative cells.

To rule out the possibility that FOSL1 upregulation was mediated by a non-cell autonomous mechanism, its expression was assessed in lung cancer cell lines derived from different mouse models^2^,^30^,^31^,^32^. *Fosl1* expression was detected in mutant *Kras* LAC cell lines but not in normal lung tissue or wild-type *Kras* squamous cell carcinoma cells (Fig. 2i and Supplementary Fig. 2g). Notably, FOSL1 expression was preserved in cell lines derived from metastatic sites^32^. These results indicate that FOSL1 is upregulated in an autochtonous model of *Kras*-driven LAC by a cell-autonomous mechanism and suggest that it may be important in lung tumorigenesis, including advanced metastatic stage.

### Preferential sensitivity of mutant KRAS cells to FOSL1 loss

**Figure 3:**
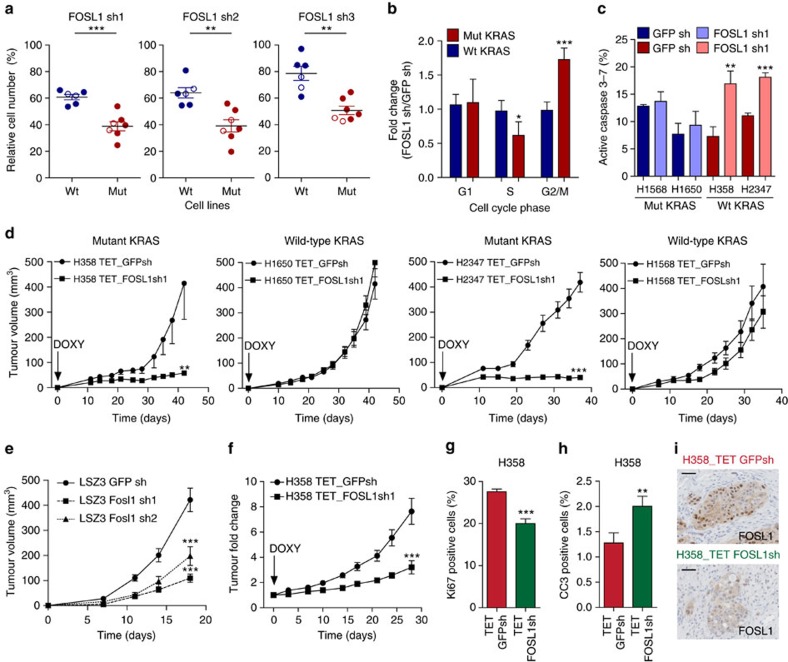
Impact of FOSL1 inhibition on LAC cell proliferation *in vitro* and *in vivo*. (**a**) Percent cell viability of human wild-type (HCC78, H1437, H1568, H1650, H1703 and H2126) and mutant (H23, H358, H441, A549, H1792, H2009 and H2347) KRAS LAC cell lines with three independent FOSL1 shRNAs. Data are relative to number of cells expressing a GFP shRNA. Relative cell number was assessed by an MTS assay for 4 days and repeated three times. (**b**) Cell cycle analysis using an EdU incorporation assay of mutant (H358 and H2347) and wild-type (H1568 and H1650) KRAS cell lines expressing an inducible shRNA against GFP or FOSL1. shRNA expression was induced by doxycycline (1 μg ml^−1^) for 3 days and then cells plated for 24 h before analysis. Data are normalized to cells expressing a GFP shRNA. Result is average of three independent experiments. Error bars correspond to s.d. (**c**) Analysis of active caspase 3/7 in the same cells as in (**b**). Data are normalized to cells expressing a GFP shRNA. Result is average of three independent experiments. Error bars correspond to s.d. (**d**) Average tumour volume of xenografts from H358, H2347, H1568 and H1650 cells expressing an inducible GFP or FOSL1 shRNA (*n*=8–12 per group). Mice were fed 2 mg ml^−1^ doxycycline plus 5% sucrose in drinking water when cells were implanted. Error bars correspond to s.e.m. (**e**) Average tumour volume of xenografts from mouse LAC cells (LSZ3) transduced with a control GPF shRNA (circle) and two shRNAs (square and triangle) against *Fosl1* (*n*=8 per group). Error bars correspond to s.e.m. (**f**) Average tumour volume change of xenografts from H358 cells with inducible GFP and FOSL1 shRNAs (*n*=12 per group) relative to start of doxycycline treatment. Doxycycline treatment (2 mg ml^−1^ doxycycline plus 5% sucrose in drinking water) started at day 18 when tumours had an average volume of 80–100 mm^3^. Error bars correspond to s.e.m. ***P<0.001. (**g**) Analysis of Ki67 positive cells in representative areas (*n*=15 per group) of tumours in (**f**). Error bars correspond to s.e.m. ***P<0.001. (**h**) Analysis of cleaved caspase 3 positive cells in representative areas (*n*=10 per group) of tumours in (**f**). Error bars correspond to s.e.m. *P* value obtained using a Student's *t*-test. *P<0.05; **P<0.01. ***P<0.001. (**i**) Immunohistochemistry to detect FOSL1 expression in representative sections of the same tumours as in (**f**). Scale bars 50 μm.

To test the functional relevance of FOSL1 in KRAS-driven LAC, FOSL1 was depleted by interference RNA in mutant and wild-type *RAS* cell lines (Supplementary Fig. 3a). Mutant *RAS* cells were significantly more sensitive to FOSL1 inhibition than wild type for three independent shRNAs, suggesting an on-target effect (Fig. 3a). This result was consistent among mutant and wild-type cell lines with similar population doubling (Fig. 3a, open circles, and Supplementary Fig. 3b) suggesting that the sensitivity to FOSL1 loss is specific to *KRAS* genotype. Moreover, the effect of FOSL1 inhibition was extended to mutant *RAS* cells from large cell carcinoma (Supplementary Fig. 3c). Likewise, FOSL1 depletion in two mouse LAC cell lines derived from a *Kras*-driven mouse model led to significant decrease in cell viability (Supplementary Fig. 3d). Next, the cellular mechanisms underlying the effects of FOSL1 loss were analysed using an inducible shRNA that compromised proliferation of mutant *KRAS* cells *in vitro* as seen for the same constitutive shRNA (Supplementary Fig. 3e,f). FOSL1 loss led to shortened S-phase, increased G2 phase and induction of apoptosis (Fig. 3b,c).

Then, we asked whether FOSL1 would be required for LAC tumorigenesis. Injection of human mutant *KRAS* cells infected with the inducible FOSL1 shRNA into immunodeficient mice treated with doxycycline yielded tumours of significant smaller volume than control cells (Fig. 3d and Supplementary Fig. 3g). These differences were due to a significant decrease in the number of proliferating cells and an increase in apoptotic cells in concordance with reduced FOSL1 expression (Supplementary Fig. 3i–k). Conversely, average tumour volume of KRAS wild-type cells with FOSL1 inhibition did not change compared to the control group (Fig. 3d and Supplementary Fig. 3h). Likewise, FOSL1 depletion in mouse *Kras*-driven lung cancer cells decreased tumour volume (Fig. 3e and Supplementary Fig. 3l).

To investigate whether FOSL1 was also important in tumour maintenance, mutant *KRAS* cells were engrafted and grown until average tumour diameter reached 80–100 mm^3^ prior to doxycycline administration. FOSL1 inhibition significantly decreased tumour fold change compared to control shRNA (Fig. 3f and Supplementary Fig. 3m). These changes correlated with less Ki67 and more cleaved-caspase 3 positive cells in FOSL1-depleted tumours (Fig. 3g–i). All in all, these data show that FOSL1 plays a role in KRAS-driven tumorigenesis.

### FOSL1 is required for mouse lung tumorigenesis

**Figure 4:**
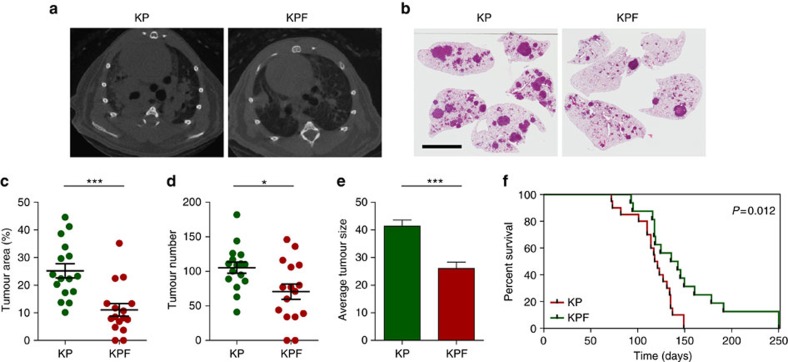
Fosl1 is required in a genetically engineered mouse model of advanced LAC. (**a**) Representative microCT scans of tumours in *Kras*^LSLG12D/+^; *Trp53*^flox/flox^; *Fosl1*^+/+^ (KP) and *Kras*^LSL-G12D/+^; *Trp53*^flox/flox^; *Fosl1*^flox/flox^ (KPF) mice treated with AdCre and allowed to develop tumours for 12 weeks. (**b**) Representative H and E stained sections of KP and KPF lungs. Scale bar is 6 mm. (**c**) Dot plot of the quantification of tumour area in KP (*n*=16) and KPF (*n*=16) groups (*n*, number of mice per group). Error bars correspond to s.e.m. *P* value obtained using a Mann–Whitney test. (**d**) Dot plot showing mean number of tumours per mouse in KP and KPF mice. Error bars correspond to s.e.m. *P* value obtained using Student's *t*-test. (**e**) Bar graph of the average of tumour size in KP and KPF groups. Error bars correspond to s.e.m. *P* values obtained using Student's *t*-test. (**f**) Kaplan–Meier plot of KP (*n*=20) and KPF (*n*=16) mice. *P* values obtained using the log-rank test (Mantel-Cox). *P<0.05; ***P<0.001.

To investigate the role of FOSL1 in a model of LAC initiation by oncogenic *Kras*, *Kras*^LSL-G12D/+^; *Trp53*^f/f^; *Fosl1*^+/+^ (KP) and *Kras*^LSL-G12D/+^; *Trp53*^f/f^; *Fosl1*^f/f^ (KPF) mice (Supplementary Fig. 4a) were intratracheally administered with adenoviral Cre (AdCre). A decrease in tumour burden was observed in KPF mice compared to KP control mice by microcomputed tomography and histology after 12 weeks of AdCre administration (Fig. 4a,b). Histological analysis revealed a significant decrease of tumour area in KPF compared to KP mice (Fig. 4c), despite the presence of large KPF tumours with incomplete recombination of *Fosl1* floxed alleles (Supplementary Fig. 4b). Analysis of total tumour number in KP vs KPF mice showed a significant decrease in the number of tumours initiated in lung epithelial cells depleted of *Fosl1* (Fig. 4d). Likewise, analysis of tumour size revealed that tumours in KPF mice were significantly smaller compared to KP (Fig. 4e). In this regard, binning of the total number of tumours into quartiles by size revealed that median differences at each quartile were statistically significant between the two groups (Supplementary Fig. 4c). Lastly, *Fosl1* ablation in KPF mice prolonged overall survival compared to control mice (Fig. 4f). These results suggest that *Fosl1* functions in initiation and progression of mutant *Kras* tumours and demonstrate an important role of FOSL1 in *Kras*-driven LAC oncogenesis *in vivo*.

### FOSL1 plays a relevant role in pancreatic adenocarcinoma

**Figure 5:**
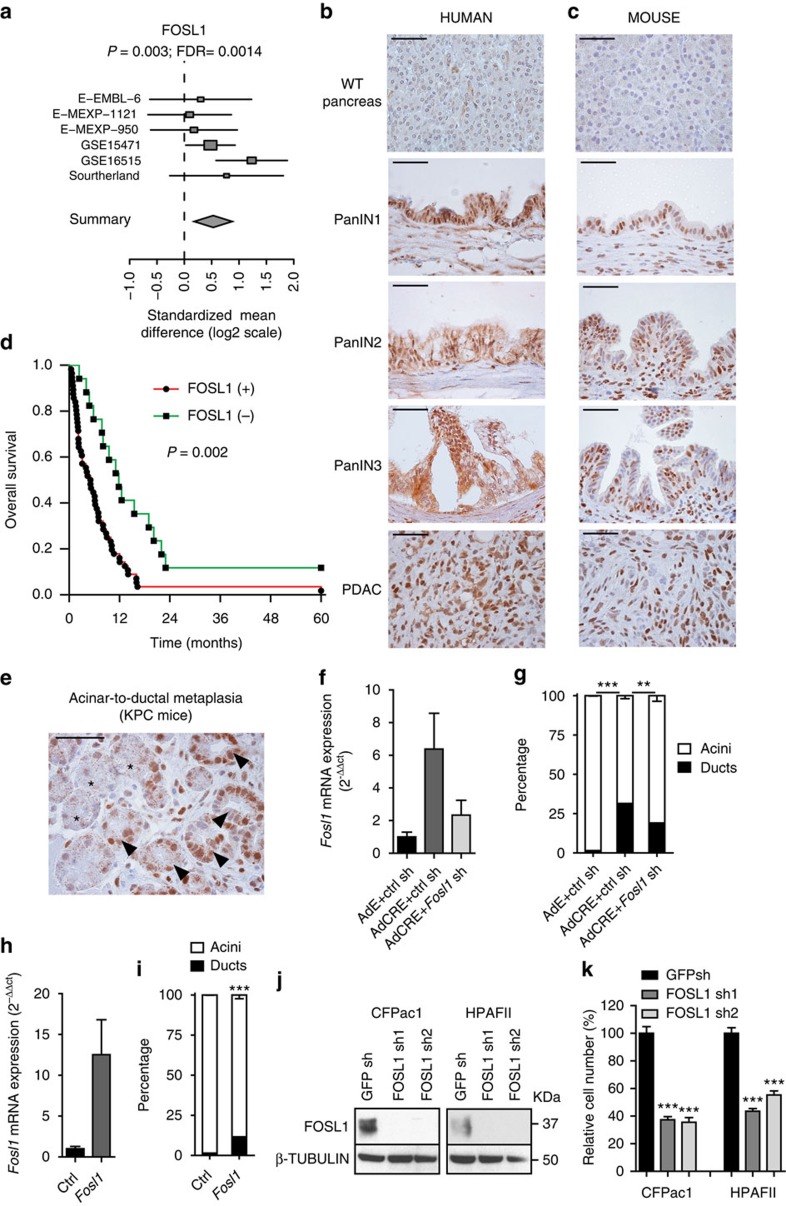
Requirement for FOSL1 expression in PDAC. (**a**) Standardized Mean Difference (log2 scale) of FOSL1 mRNA expression meta-analysis across six PDAC data sets. (**b**,**c**) FOSL1 protein expression by immunohistochemistry in normal pancreas, pancreatic intraepithelial neoplasias and PDAC of human (**b**) and mouse (**c**) tissues. Scale bars: 50 μm. (**d**) Kaplan–Meier plot of FOSL1 protein expression in PDAC patients. (**e**) Immunohistochemistry for FOSL1 detection in normal pancreas acini (stars) and ADM (arrowheads) in *p48*^cre/+^, *Kras*^loxG12D^, *Trp53*^Δ/Δ^ (KPC) mice. (**f**) *Fosl1* mRNA expression in acinar cells from *Kras*^loxG12D^ mice treated with control adenovirus (AdE), adenovirus Cre (AdCre) and AdCre plus a shRNA targeting *Fosl1*. (**g**) Percentage of acinar (white) and ductal (black) clusters in the same ADM experiment as in (**f**). Two independent organoid cultures were seeded in triplicate. After 5 days in culture, three areas were counted at random in each triplicate. (**h**) *Fosl1* mRNA expression in acinar cells from *Kras*^+/+^ mice expressing an empty (ctrl) and a *Fosl1*-expressing vector. (**i**) Percentage of acinar (white) and ductal (black) clusters in the same ADM experiment as in (**h**). Two independent organoid cultures were seeded in triplicate. After 5 days in culture, three areas were counted at random in each triplicate. (**j**) Western blot analysis of FOSL1 protein in mutant *KRAS* PDAC cells (CFPac1 and HPAFII) transduced with two independent *FOSL1* shRNAs. (**k**) Relative cell number assessed by MTS of mutant *KRAS* PDAC cells upon FOSL1 inhibition. Result is representative of three independent experiments. Error bars correspond to s.d. *P* value obtained using Student's *t*-test. **P<0.01; ***P<0.001.

The identification of FOSL1 through a cross-tumours approach including LAC, PDAC, CRC, CCA and MM suggested that it could be relevant in other KRAS-driven tumours. Thus, we investigated if FOSL1 played a role in PDAC where *KRAS* stands as a prominently mutated oncogene. A significant upregulation of FOSL1 mRNA in pancreatic cancer tissue compared to normal was observed by meta-analysis (Fig. 5a). These findings were recapitulated at the protein level in human and mouse PDAC cell lines (Supplementary Fig. 5a,b). To rule out the possibility that FOSL1 was regulated through KRAS-independent mechanisms in PDAC cells, KRAS expression was inhibited and decreased FOSL1 expression was observed (Supplementary Fig. 5c).

To extend these findings to the clinical setting, FOSL1 expression was assessed in PDAC patients (*n*=72) (Supplementary Table 3). Antibody specificity was validated in human cancer cells using FOSL1 shRNAs (Supplementary Fig. 5d). No expression was observed in normal pancreas tissue, whereas FOSL1 positive cells were detected in pancreatic intraepithelial neoplasias through PDAC (Fig. 5b and Supplementary Fig. 5e). Similar results were observed in a mouse model of PDAC (*p48*^+/Cre^; *Kras*^LSLG12D^, *trp53*^flox/flox^ (KPC)) (Fig. 5c and Supplementary Fig. 5e). More importantly, high FOSL1 protein expression was a marker of poor patient survival (*P*=0.002, log-rank test, Mantel-Cox) (Fig. 5d). These results were recapitulated in an independent PDAC cohort at the mRNA level (*P*=0.048, log-rank test, Mantel Cox) (Supplementary Fig. 5f). Overall, these findings highlight a strong clinical role for FOSL1 in human PDAC.

To investigate FOSL1 functional role in PDAC, we focused on two different stages of oncogenesis. First, we explored FOSL1 function in the transdifferentiation of mouse acinar cells into duct-like cells (acinar-to-ductal metaplasia, ADM) upon mutant *Kras* expression^33^. We observed FOSL1 upregulation in ADM of sections from KPC mice (Fig. 5e) and in a mouse *ex-vivo* ADM assay of *Kras*^LSLG12D^ acini^34^ (Fig. 5f). In the later, *Fosl1* upregulation was accompanied by upregulation of the ductal marker *Ck19* (Supplementary Fig. 5g). Subsequent *Fosl1* knockdown studies revealed FOSL1 requirement for efficient duct formation of acinar cells upon *Kras* activation (Fig. 5g and Supplementary Fig. 5g). Lastly, *Fosl1* overexpression was sufficient to promote ADM, albeit at lower levels than *Kras* activation (Fig. 5h,i and Supplementary Fig. 5h). These observations suggest that FOSL1 plays a role in the development of PDAC.

Second, we studied whether FOSL1 is a gene required for PDAC. Inhibition of FOSL1 expression in two KRAS-dependent PDAC cell lines showed a significant decrease in cell viability (Fig. 5j,k), suggesting that FOSL1 is important in homeostasis of PDAC cells. Collectively, these observations underline a relevant role of FOSL1 in different stages of PDAC oncogenesis.

### FOSL1 regulates a gene signature that predicts poor survival

**Figure 6:**
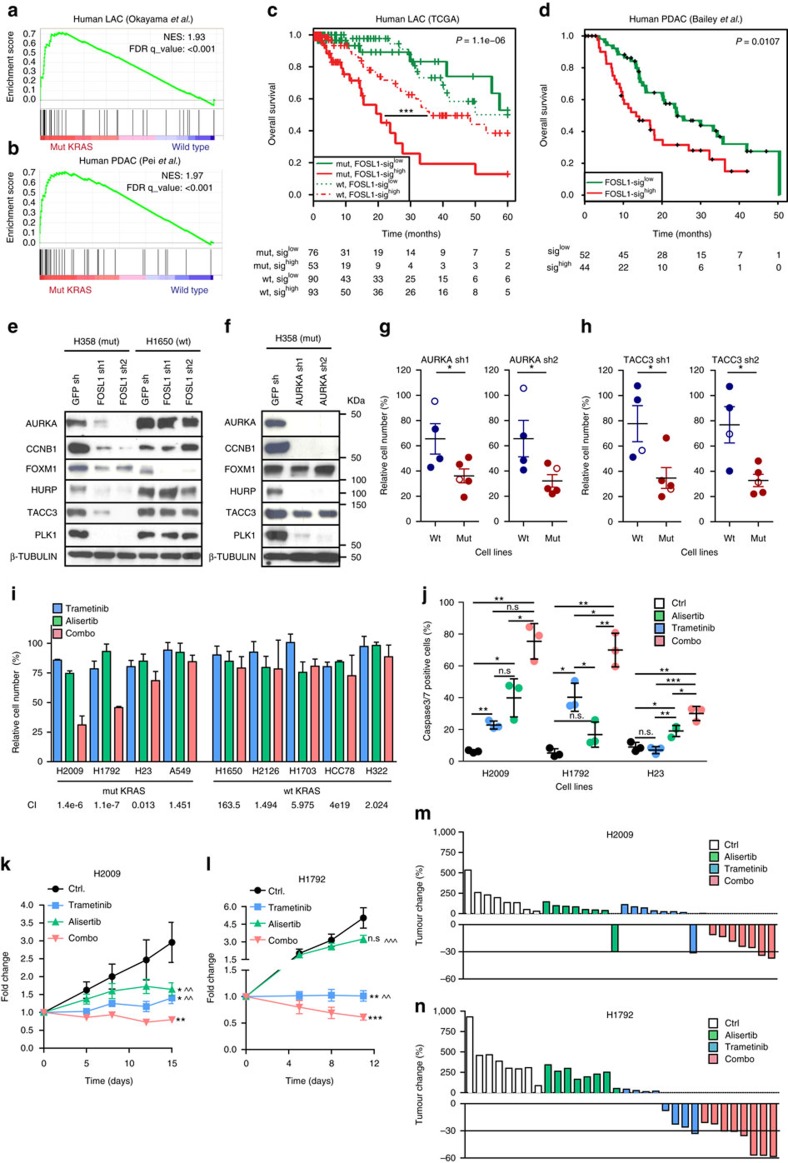
FOSL1 regulates a transcriptional program including genes involved in mitosis progression amenable to pharmacological inhibition. (**a**,**b**) GSEA of human LAC (**a**) and PDAC (**b**) data sets comparing mutant *KRAS* patients to wild-type *KRAS* patients. (**c**) Survival analysis of LAC patients (TCGA data set) stratified by *KRAS* status and expression of a FOSL1 signature. (**d**) Survival analysis of PDAC patients stratified by expression of a FOSL1 signature. *P* values obtained using the log-rank test (Mantel-Cox). (**e**) Western blot analysis on the indicated mitotic genes in mutant (H358) and wild-type (H1650) *KRAS* LAC cells after FOSL1 inhibition by two independent shRNAs. Western blot is representative of three independent western blots with different lysates. (**f**) Western blot analysis on the indicated mitotic genes in mutant *KRAS* LAC cells (H358) after AURKA inhibition by two independent shRNAs. Western blot is representative of three independent western blots with different lysates. (**g**,**h**) MTS assay of wild-type (HCC78, H1437, H1650 and H2126) and mutant *KRAS* (H23, H358, A539, H2009 and H2347) treated with two independent shRNAs targeting AURKA (**g**) or TACC3 (**h**). Open circles represent cell lines with similar population doubling time. Error bars correspond to s.d. Assay is average of two independent experiments. (**i**) MTS analysis of mutant and wild-type *KRAS* cells lines treated with alisertib (500 nM), trametinib (500 nM) or both. CI: combination index. CI<1 in bold. Results are average of four different independent treatment experiments performed in triplicate. (**j**) Analysis of active caspase 3/7 cells in H2009, H1792 and H23 cells treated with vehicle, alisertib (1 μM), trametinib (1 μM) or both for 72 h. *P* values obtained using Student's *t*-test. (**k**,**l**) Analysis of tumour volume of mice injected with H2009 or H1792 cell lines and orally administered vehicle, alisertib (25 mg kg^−1^), trametinib (1 mg kg^−1^) or both. Tumours were grown until average tumour volume ranged from 80 to 100 mm^3^ and randomized before treatment starts. Error bars correspond to s.e.m (*n*=8 per group). Comparisons to control group: *P<0.05; **P<0.01; ***P<0.001. Comparisons to combo group: ^^*P*<0.01; ^^^*P*<0.001. *P* values obtained using Student's *t*-test. (**m**,**n**) Analysis of tumour change from samples in (**i**,**j**) at the end of each experiment (*n*=8 per group).

To explore the molecular mechanisms elicited by FOSL1, transcriptomic profiling was performed on mutant *KRAS* LAC cells. Forty-five genes were downregulated upon FOSL1 inhibition (FC<0.5 and B>0) (Supplementary Fig. 6a). This list included genes or pathways whose inhibition had been shown to impair Kras-driven tumorigenesis such as Hexokinase 2 (ref. 35), FOXM1 (ref. 16) or PI3K/mTOR^36^. To investigate if the FOSL1 signature (FOSL1 sig) was representative of mutant *KRAS* tumours, GSEA was carried out in LAC and PDAC showing a significant enrichment in mutant *KRAS* tumours across several human and mouse data sets (Fig. 6a,b and Supplementary Fig. 6b,c). Interestingly, high expression of the FOSL1 sig was found in LAC patients harbouring *KRAS* mutations, and was associated with the poorest survival outcome (Fig. 6c). This result was recapitulated in a PDAC patient cohort^25^ (Fig. 6d).

Given FOSL1's strong clinical role, we explored the biological processes associated with the FOSL1 sig. The top biological processes identified by gene ontology (GO) with a significant enrichment in FOSL1 genes were microtubule-based process, mitotic cell cycle, regulation of response to stress, negative regulation of biological process and cell cycle phase (Supplementary Fig. 6d). We found a decrease in mRNA of genes involved in mitosis-related processes such as *AURKA*, *CCNB1*, *FOXM1*, and the AURKA targets *HURP* and *TACC3* (refs 37, 38, 39, 40) (Supplementary Fig. 6e). Of note, protein expression of AURKA, CCNB1, HURP and TACC3 was decreased upon FOSL1 inhibition in an independent mutant *KRAS* LAC cell line but not in wild-type cells with similar proliferation rate *in vitro* and *in vivo* (Fig. 6e). Additionally, the AURKA target PLK1, previously identified as a synthetic lethal interaction with KRAS^38^, was also diminished upon FOSL1 inhibition in a genotype-specific manner (Fig. 6e). Downregulation of mitotic proteins was also observed in PDAC cells at the mRNA and protein level (Supplementary Fig. 6f,g). These results strongly suggest that KRAS oncogene intersects with the mitosis machinery through FOSL1 in human cancer.

The above results suggested that the phenotype originated by FOSL1 loss involves components of the mitotic machinery. A central event in mitosis is the phosphorylation of histone H3 (HH3) by AURKA^40^. Using taxol, which interferes with mitotic spindle function and blocks cells in mitosis, we found that FOSL1 inhibition led to decreased phospho-HH3 levels in mutant *KRAS* LAC cells compared to control cells, in concordance with decreased expression of mitotic genes (Supplementary Fig. 6h,i). These findings argue that the phenotype observed in FOSL1-silenced cells may involve the regulation of components of the mitotic machinery.

### AURKA depletion recapitulates FOSL1 loss phenotype

Mutant *KRAS* tumours undergo a high degree of mitotic stress and, thus, genes contributing to this phenotype may represent cancer vulnerabilities^20^. We focused on AURKA, for which pharmacological inhibitors are currently being investigated in clinical trials. AURKA knockdown led to a profound protein decrease of the mitotic genes CCNB1, HURP and PLK1, while only a partial reduction of TACC3 and FOXM1 was observed (Fig. 6f). At the functional level, AURKA inhibition induced a decrease in the relative number of mutant *KRAS* cells compared to wild type (Fig. 6g). However, AURKA overexpression did not rescue the FOSL1-knockdown proliferative phenotype of mutant *KRAS* cells (Supplementary Fig. 6j). In this regard, we observed that AURKA overexpression was unable to recover the expression of TACC3 in the rescue experiment (Supplementary Fig. 6k). In light of these results and given that TACC3 is a potent AURKA coactivator^41^ that is only partially regulated by this mitotic kinase, we reasoned that if TACC3 is required for AURKA activity in KRAS-driven tumours its depletion would recapitulate the AURKA-inhibition phenotype. Notably, TACC3 inhibition was also preferentially detrimental to proliferation of mutant *KRAS* LAC cells (Fig. 6h and Supplementary Fig. 6l).

Next, survival analyses were carried out in two independent LAC and PDAC patient cohorts. We observed that high levels of *AURKA* were a marker of poor prognosis in both tumour types (Supplementary Fig. 6m), highlighting a clinical role for this kinase in KRAS-driven tumours.

Based on the AURKA knockdown results and the fact that its upstream regulator FOSL1 can be regulated by different KRAS effectors, including the MEK-ERK pathway, we posited that combined inactivation of AURKA and MEK would be more deleterious to mutant *KRAS* cells than single protein inactivation. Pharmacological inhibition of AURKA (alisertib) and MEK1/2 (trametinib) was carried out in mutant and wild-type LAC cell lines using drug concentrations equal or lower than the GI25 by MTS analysis (500 nM and 1,000 nM), which achieved complete protein inactivation after a 3-day treatment (Supplementary Fig. 6n,o). Analysis of the dual inhibition revealed a synergistic effect in three out of four mutant *KRAS* cell lines for all possible drug combinations, while no synergy was observed in wild-type *KRAS* cell lines (Fig. 6i and Supplementary Fig. 6p). The synergistic effect correlated with a significantly higher induction of active caspase 3/7 positive cells in the inhibitor combination compared to single treatments (Fig. 6j).

The *in vitro* results prompted us to test the effect of alisertib and trametinib combination *in vivo.* Mutant *KRAS* tumours were treated for 14 days (H2009) or until control tumours reached the largest volume allowed under the ethical protocol (11 days, H1792). Alisertib or trametinib induced significant reduction of tumour volume in the two xenograft models. However, concomitant treatment induced a more significant reduction than each single drug (Fig. 6k,l). Of note, the drug combination led to regression of 15 out of 16 tumours in the two models while alisertib and trametinib alone to one and five tumours, respectively (Fig. 6m,n). Furthermore, the different treatments had no impact on mice weight (Supplementary Fig. 6q). Collectively, these results support a functional role of the FOSL1 target AURKA in KRAS-driven tumours and unveil the dual inhibition of AURKA and MEK activation as a potential strategy to treat tumours with *KRAS* mutations.

## Discussion

We described an integrative gene expression-based screen to unveil KRAS dependencies. Our approach followed a two-tiered ‘zoom-in' strategy to first identify KRAS-regulated candidate genes by a cross-species meta-analysis of laboratory data and, second, to select genes frequently upregulated across human KRAS-driven cancers by querying the 19 KRAS candidate genes against a panel of five different tumour types. Selection within the KRAS eight-gene cross-tumours signature of the candidate gene FOSL1 for follow-up studies relied on the incorporation of patient outcome information. The fact that loss-of-function experiments demonstrated that *FOSL1* is important in mutant *KRAS* LAC argues that the overall approach integrating gene-expression and survival data is successful to identify genes with a relevant role in KRAS-driven cancer. Moreover, the survival and genetic data were recapitulated in PDAC, what supports the analysis across different tumour types to unveil common KRAS targets.

Inhibition of early events in the phylogeny of a tumour could attenuate tumour growth and relapse^42^. However, intratumour heterogeneity arises as a hurdle for the identification of such events, thus favouring tumour progression and relapse. One potential strategy to unveil those early events relies on the study of initial stages of tumorigenesis. In this regard, our strategy using experimental systems that represented initial stages of KRAS-induced cell transformation and tumour progression yielded genes with a role in advanced disease such as FOSL1. These results are in tune with recent findings that took advantage of a gene expression signature derived from mouse lung hyperplasias driven by oncogenic *Kras* to uncover DDR1 as a therapeutic target in KRAS-driven LAC^43^.

In the process of RAS transformation, FOSL1 was originally considered a gene involved in controlling G1/S phase transition by upregulating CCND1 (ref. 27). However, FOSL1 inhibition experiments later unveiled a role in the control of a motility and invasion programme in human colon cancer cells expressing oncogenic KRAS^G13D^ (refs 29, 44) *in vitro*. Our study provides evidence that, under endogenous KRAS oncogene expression, FOSL1 can as well regulate a set of genes involved in mitosis progression, a cellular function previously proposed to work orthogonally to KRAS oncogene in human cancer^20^,^22^ but for which a direct link to the KRAS oncogene network was missing. Among the identified genes, the AURKA targets and coactivators *TACC3* and *HURP* had not been previously linked to the KRAS signalling network. On the other hand, *AURKA* was found upregulated in PDAC^45^,^46^ and malignant peripheral nerve sheath sarcomas with abnormal RAS signalling^47^, and reported to phosphorylate the KRAS effectors RalGDS and RalA when ectopically expressed in mouse and human cells^48^,^49^. However, the molecular mechanisms whereby oncogenic KRAS regulated AURKA expression and its clinical and functional role in KRAS-driven tumours were largely unknown. Our data also revealed that the mitosis kinase *PLK1* is regulated by FOSL1, shedding light into the molecular mechanisms regulating this kinase in the context of KRAS oncogene. All in all, these observations indicate that FOSL1 links KRAS oncogene to genes involved in mitotic fitness, and suggest that AURKA and TACC3 may partially mediate the ‘synthetic sensitivity' of mutant *KRAS* tumours to FOSL1 loss.

Although FOSL1 inhibition involved the regulation of mitotic genes, sensitivity of mutant *KRAS* cells to FOSL1 loss seems to occur irrespectively of the proliferative rate of tumour cells. This is supported by the fact that both *in vitro* and *in vivo* mutant *KRAS* cells showed a higher sensitivity to FOSL1 knockdown than wild-type cell lines with similar population doubling. A possible explanation of the ‘synthetic sensitivity' to FOSL1 loss maybe the heightened mitotic stress reported in mutant *KRAS* cells^20^. Nonetheless, despite the central role of the mitotic machinery to mutant KRAS phenotype, the contribution of other members of the FOSL1 signature to the effect induced by FOSL1 loss remains unexplored and may also help to explain the impact of FOSL1 inhibition in homeostasis of mutant *KRAS* tumours.

Our findings also argue that a better understanding of the molecular events controlled by prominent transcriptional nodes of the KRAS oncogene network, such as FOSL1, may expose alternative strategies to direct inhibition of transcription factors. We show that, at least for LAC, concomitant inhibition of the FOSL1 target AURKA and the KRAS canonical effector MEK is more effective than single treatments. This result provides the rationale for novel therapeutic approaches against tumours with *KRAS* mutations and adds to recently reported combinatorial strategies for the treatment of KRAS-driven tumours involving MEK inhibitors (MEKi)^50^ or inhibitors targeting central kinases of mitotic progression, such as PLK1 (ref. 51). Clinical trials combining alisertib and targeted therapies such as the epidermal growth factor receptor (EGFR) inhibitor (EGFRi) erlotinib, (NCT01471964), the pan-RAFi MLN0128 (NCT02327169), the mTORi MLN1117 (NCT02551055) and sapanisertib (NCT02719691) are being conducted in solid tumours, thus paving the path for combination studies with MEKi in KRAS-driven tumours.

In summary, we developed an integrative analysis for the identification of KRAS oncogene vulnerabilities that unveiled opportunities for therapeutic intervention. This general approach integrating mutation, gene-expression and survival data may be applicable to other cancers driven by not actionable oncogenes and/or tumour suppressors for which similar information is available.

## Methods

### Reagents

shRNAs to *FOSL1*: sh1–TRCN0000019541, sh2–TRCN0000019543, sh3–TRCN0000019539. *FOSL1* sh1 was also cloned into a TET inducible version of pLKO.1 vector^52^. shRNAs to *Fosl1*: sh1-TRCN0000042683, sh2-TRCN0000042686. shRNAs to AURKA: sh1–TRCN0000000656, sh2–TRCN0000000657. shRNAs to TACC3: sh1–TRCN0000290485, sh2–TRCN0000308273. Control shRNA to green fluorescence protein (GFP) has been previously described^15^. pDONR223-AURKA was a gift from William Hahn & David Root (Addgene plasmid # 23532). Pharmacological inhibitors: U0126, SP600125, LY294002 and SB203850 were from Sigma; BIX02189 was from Tocris; trametinib and alisertib were from Selleckchem.

### Lentiviral infections

Lentivirus was produced by transfection into 293FT cells as previously described^15^, filtered and applied directly to cells for infection at an MOI lower than 1.

### Quantitative RT-PCR (qRT-PCR) analysis

RNA analysis was carried out as previously published^15^.

### Western blot

Cells were scraped and lysed in buffer containing 1% NP-40, 150 mM NaCl, 50 mM Tris pH 7.4, 1 mM EDTA, 1% glycerol, supplemented with protease inhibitor cocktail (Roche), 25 mM sodium fluoride and 1 mM sodium orthovanadate. Protein samples were resolved by SDS PAGE and transferred to nitrocellulose membranes (BioRad) and incubated in 5% milk TBS-T for 1 h prior to addition of primary antibody. Antibodies used were: FOSL1 (1:2,000, #5281, Cell Signaling Technology (CST)), p-FOSL1 (1:1,000, #5841, CST), C-FOS (1:1,1000, #2250, CST), C-JUN (1:1,000, #9165, CST), JUNB (1:1,000, #3753, CST), JUND (1:1,000, #5000, CST), FOSB (1:1,000, #2251, CST), p-AURKA (1:1,000, #2914, CST), p-ERK (1:1,000, #9101, CST), ERK (1:1,000, #9102, CST), FOSL1 (1:500, sc-376148, Santa Cruz Biotechnology (SCB)), KRAS (1:500, sc-30, SCB), AURKA (1:500, sc-56881, SCB), CCNB1 (1:500, sc-245, SCB), FOXM1 (1:500, sc-376471, CST), HURP (1:500, sc-377004, SCB), KIF20A (1:500, sc-374508, SCB), TACC3 (1:1000, sc-376900, SCB), PLK1 (1:500, sc-17783), β-tubulin (1:2,000, sc-9104, SCB) and FOSL2 (1:1,000, WH0002355M1, Sigma).

Uncropped scans of the most relevant blots are provided as Supplementary Figs (7–9) in the Supplementary Information.

### Immunohistochemistry (mouse and human LAC and PDAC)

Tissues were dewaxed, hydrated and incubated in a Pascal Pressure Chamber containing Tris-EDTA (10 mM/1 mM), pH 9, at 95 °C for 30 min. Temperature was monitored using Pascal quality strips (S2800, Dako). Next, endogenous peroxidase was blocked with 3% H_2_O_2_ in water for 12 min. Slides were then incubated with a primary antibody to human and mouse FOSL1 (sc-376148, SCB, human: 1:200; mouse: 1:500) or Ki67 (1:100, RM-9106 (SP6), Thermo) at 4 °C overnight. The reaction was developed using an anti-mouse EnVision kit (K4007, Dako) and tissues counterstained with Harris hematoxylin. Validation of mouse antibody for human and mouse samples was done by western blot using two independent shRNAs against human and mouse FOSL1.

Immunohistochemistry score was blinded to researchers analysing the different experiments (that is, sample name was coded to a random number).

### Cell proliferation assay

Cell proliferation was assessed using the The CellTiter 96 AQueous Non-radioactive Cell Proliferation Assay, MTS (Promega). Experiments were read on the indicated days according to manufacturer's instructions. Data were normalized to day 0 of experiment.

### Population doubling time

Cells were counted at seeding, plated at a density of 400,000 cells per 60 mm plate and allowed to grow to a confluence of 80–90%. Then cells were harvested and total number was determined. Population doubling time was calculated using the following formula: Number of hours from seeding to harvest=[((logN(t)−logN(t0))/log_2_]. N(t) is the number of cells at time of passage and N(t0) is the number of cells seeded at previous passage.

### Cell cycle assay

Cell cycle analysis was carried out with Click-iT EdU Flow Cytometry Assay Kit (Invitrogen). Cells were seeded and maintained in culture for 24 or 48 h. At the time points indicated, cells were incubated with 10 μM EdU (5-ethynyl-2′-deoxyuridine) for 2 h. Next, cells were harvested, washed in DPBS containing 1% BSA and fixed in formaldehyde (Click-iT fixative) for 15 min at room temperature. Cells were washed in DPBS containing 1% BSA to remove formaldehyde, and permeabilized in 1X Click-iT saponin-based permeabilization and wash reagent for 15 min at room temperature. Next, cells were incubated for 30 min at room temperature in the dark, with the Click-iT reaction cocktail. After a washing step with 1X Click-iT saponin-based permeabilization and wash reagent, cells were incubated with 0.2 μg μl^−1^ RNase A (Sigma-Aldrich) for 1 h at room temperature, in the dark. 7AAD was added to the tubes 10 min before the acquisition of cells in a FACSCanto II cytometer (BD Biosciences). Data were analysed using FlowJo software v9.3.

### Apoptosis assay

Basal apoptotic levels were determined with CellEvent Caspase-3/7 Green Flow Cytometry Assay Kit (Invitrogen). After shRNA or inhibitor treatment, cells were harvested, washed and resuspended in DPBS containing 2% BSA. Next, cells were incubated with CellEvent Caspase-3/7 Green Detection Reagent for 30 min at 37 °C, in the dark. During the final 5 min of staining, 1 mM SYTOX AADvanced dead cell stain was added to the samples. Cells were acquired in a FACSCanto II cytometer (BD Biosciences) and data were analysed using FlowJo software v9.3.

### Phospho-histone H3 labelling

Cells were maintained in culture with no treatment or stimulated with 0.5 μM taxol (Sigma) for 20 h. Harvested cells were fixed and permeabilized in 70% ethanol at 4°C for 2 h. After a washing step, cells were resuspended in PBS+1% BSA at 10^6^ cells ml^−1^. Cells were incubated with phospho-histone H3 (Ser10) antibody (Cell Signaling) at 1:70 dilution for 20 min, washed and subsequently incubated with Alexa Fluor 488 fluorochrome-coupled secondary antibody (Invitrogen) at 20 μg ml^−1^ for 20 min. After washing with PBS+1% BSA, cells were incubated with 7AAD (BD Biosciences) for 10 min. Cells were acquired in a FACSCalibur cytometer (BD Biosciences) and data were analysed using FlowJo software v9.3.

### Mouse work

All experiments in mice were performed according to the institutional Animal Care Committee of the University of Navarra under the protocols CEEA/120-13 and CEEA/021-14 approved by the regional Government of Navarra. Sample size was chosen using biomath (http://www.biomath.info/power/ttest.htm) or based on similar experiments previously published by the authors. For a two-sample *t*-test, power was estimated as follows: with a sample size of 12 mice, significant differences of 30% can be detected with 80% power at a 0.05 significance level, assuming a standard deviation within groups of 0.25.

For xenograft experiments, 25 × 10^3^ (LKR13) or 2 × 10^6^ (H358, H2347, H1568 and H1650, H2009 and H1792) cells infected with specified shRNAs were suspended in 200 μl of serum-free medium and injected subcutaneously into the two lower flanks of immune-deficient 8–12-weeks-old Rag2^−/−^ and Balb/c^nu/nu^ mice (for engraftment of human and mouse cells, respectively) (Charles River). For experiments with immune-deficient mice, mice were randomized by age. One week post-injection, tumour dimensions were measured every 3 days and tumour volume was calculated using the formula: Volume= π/6 × length × width^2^. Investigator analysis was blinded (sample was coded to a random number).

For experiments with pharmacological inhibitors, alisertib (25 mg kg^−1^), trametinib (1 mg kg^−1^) or dual administration was done by oral gavage daily. Mice were randomized by tumour size to minimize tumour volume differences among the four groups.

For mouse genetics experiments, mice were intercrossed to generate *Kras*^LSL-G12D/+^; *Trp53*^f/f^; *Fosl1*^+/+^ and *Kras*^LSL-G12D/+^; *Trp53*^f/f^; *Fosl1*^f/f^. Mice were in a mixed 129/Sv and C57bl/6 background. Genotyping of mice was done on DNA extracted from tail clippings as described previously^53^,^54^. For induction of lung tumours, mice were intratracheally administered a dose of 1 × 10^7^ p.f.u. of AdCre and lungs harvested 12 weeks after infection. Blind analyses were carried out when tumour burden, tumour number or tumour size was scored in the genetic experiment. Score of immunohistochemistry analyses was also blinded.

### MicroCT scanning and histology analysis

Mice were anesthetized with 2% isoflurane and scanned using a GE Healthcare microCT scanner. Analysis of tumour burden, number of tumours and tumour size was carried out on H/E stained sections of mouse lung as previously described^15^.

### Acinar-to-ductal metaplasia (ADM) assay

ADM assays were done as previously described^18^.

### Drug combination studies

Mutant and wild-type KRAS cell lines were plated at density ranging from 4,000 to 10,000 cells in 96-well plates and treated on the following day with single drugs or both (*n*=6 wells per condition). The combination index (CI) was obtained using the CompuSyn software (www.combosyn.com), which takes advantage of the Chou-Talalay method for drug combination^55^. This method is based on the median-effect equation, derived from the mass-action law principle, which is the unified theory that provides the common link between single entity and multiple entities, and first-order and higher-order dynamics. The resulting CI offers quantitative definition for additive effect (CI=1), synergism (CI<1) and antagonism (CI>1) in drug combinations. For the determination of the CI, at least two working concentrations for each drug are required. 1 and 0.5 μM of alisertib and trametinib were used to obtain the CI for each cell line.

### Cell lines

Mouse lung cancer cell lines LKR10, LKR13 (Julien Sage), LSZ1, LSZ2, LSZ3, LSZ5 (Silve Vicent), 389N1 and 482N1 (Chen-Hua Chuang), and immortalized normal pancreas epithelial cells, KF07, were grown in DMEM supplemented with 10% FBS and 1% penicillin-streptomycin. Human non-small cell lung cancer and pancreatic cancer lines used had either wild-type *RAS* alleles (NCI-HCC78, NCI-H322, NCI-H1395, NCI-H1437, NCI-H1568, NCI-H1650, NCI-H1703 and NCI-H2126) or were mutant for *RAS* (NCI-H23, NCI-H358, NCI-H441, NCI-H460, NCI-A549, NCI-H1299, NCI-H1792, NCI-H2009, NCI-H2087, NCI-H2347, CFPac1 and HPAFII). Human cell lines were from ATCC. Human cancer cell lines were authenticated by the Genomics Unit at CIMA using Short Tandem Repeat profiling (AmpFLSTR Identifiler Plus PCR Amplification Kit) and grown according to ATCC specifications. All cell lines were tested using the MycoAlert Mycoplasma Detection Kit (LONZA). Only mycoplasma negative cells were used.

### Patient-derived xenografts

Patient-derived xenografts were obtained as previously described^56^.

### Human patients

An informed consent from patients was obtained for all human samples analysed in this study.

### Transcriptome analysis

Samples of the experiments used for KRAS gene signature selection (Sweet-Cordero *et al*.^9^, GSE15325 and GSE17671) were normalized with robust multi-array average (RMA)^57^ and after quality assessment and outlier detection with R/Bioconductor^58^, one of the mouse samples was considered an outlier and discarded along with its corresponding control. In a filtering process probe sets with an expression value lower than 5 in more than the 50% of the samples of all the studied conditions (KRAS_mut and KRAS_wt) were considered as not expressed in the experiment under study. LIMMA (Linear Models for Microarray Data)^59^ was used to identify the probe sets with significant differential expression between experimental conditions. Genes were selected as significant using a B cutoff B>0. The KRAS gene set was defined as the genes with B>0 and logFC>1 in at least two of the three studied experimental models.

Enrichment of the obtained gene set in human patients with mutated KRAS (TCGA LAC, Chitale *et al*.^60^, GSE31210, GSE15471, GSE42284, GSE3225, TCGA MML and GSE36133) or mouse models (GSE15326 and GSE32277) was analysed with GSEA^61^. Public datasets used for this analysis were downloaded from Gene Expression Omnibus (GEO) data repository (http://www.ncbi.nlm.nih.gov/geo) or TCGA Data Portal (https://tcga-data.nci.nih.gov/tcga/tcgaHome2.jsp). TCGA processed data for RNA-Seq experiments of LAC samples and microarray raw data for acute myeloid leukemia samples were downloaded for the logFC calculation of the comparison KRAS_mut vs KRAS_wt. Microarray raw data was also downloaded from GEO, normalized with RMA^57^ and analysed for the logFC calculation using R^58^. In the case of the identification of FOSL1 transcriptional targets in mutant KRAS LAC cells, microarrays were normalized with RMA^57^. Quality assessment, outlier detection and filtering process of probesets with an expression value lower than 4 in more than the 50% of the samples were performed with R^57^. LIMMA^59^ was used to identify the probe sets with significant differential (B>0). Data of microarray analysis on H2009 transduced with FOSL1 shRNA are publicly available in GEO database with the accession number GSE76290.

Hierarchical clustering of microarray data was performed with R^57^ and functional enrichment analysis of Gene Ontology categories^62^ was performed using the hypergeometric distribution in R^57^.

Meta-analysis of PDAC data sets was done as previously described^18^,^63^.

For Kras status predictor, a machine learning algorithm based on logistic regression^64^ was applied to public datasets (GSE12667, GSE16515, GSE26566, GSE26939 and Battacharjee *et al*.^65^) in order to distinguish patients with mutated KRAS from patients with wt KRAS with the selected eight-gene signature summation^66^. The performance of each classifier was evaluated using Receiver Operator Characteristics Curve analysis^67^. Both the classifiers and the corresponding Receiver Operator Characteristics analyses were performed using R^58^. The same methodology was applied to patients or cell lines with mutated EGFR (GSE31210, Nguyen *et al*.^68^ and GSE36133) and LAC patients of the TCGA dataset with mutations in EGFR, BRAF or DDR2, EML-ALK4 fusions, or MYC amplification plus no KRAS mutation (https://tcga-data.nci.nih.gov/tcga/tcgaHome2.jsp).

Survival analysis was conducted on both gene sets and individual genes using TCGA LAC data (https://tcga-data.nci.nih.gov/tcga/tcgaHome2.jsp). Log-rank test was used to calculate the statistical significance of differences observed among Kaplan–Meier curves^69^. In the case of the gene sets, a summation of all the genes for a particular sample was calculated as previously described^66^. Multivariate Cox proportional hazards analysis was also performed considering the age, gender, tumour stage and the expression of each gene set or individual gene as covariates^70^. All the survival analyses were performed with R^58^ and *P* values<0.05 were considered statistically significant. Survival analysis on KRAS eight-gene signature and FOSL1 47-gene signature using TCGA breast invasive carcinoma (BRCA) and lung squamous cell carcinoma (LUSC) RNA-seq data (https://tcga-data.nci.nih.gov/tcga/tcgaHome2.jsp) was performed as described for the TCGA LAC data.

### Statistical analyses

Sample size was chosen using http://www.biomath.info/power/ttest.htm or based on similar experiments previously published by the authors. For comparison of two groups, samples were explored for normality (Shapiro–Wilk test) and variance (Levene test). Groups with normal distribution of samples followed a *t*-test. Non-normal samples were analysed using a Mann–Whitney test (equal variances) or a Median test (unequal variances). All analyses were two-tailed. Error bars correspond to either standard deviation (s.d.) or standard error of the mean (s.e.m) as indicated for each experiment. Significant *P* values in the text and graphs correspond to <0.05 (*), <0.01 (**) or <0.001 (***). Statistical analyses were done using SPSS software.

### Primer sequences for qRT-PCR


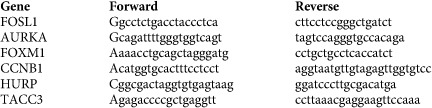


### Data availability

The datasets generated and/or analysed during the current study are available through GEO or the corresponding references.

KRAS gene signature selection: GSE49200, GSE15325 and GSE17671.

Enrichment of KRAS-dependent candidates in human patients: TCGA LAC data set, Chitale *et al*.^60^ (http://cbio.mskcc.org/Public/lung_array_data/), GSE31210, GSE15471, GSE42284, GSE3225, TCGA MML data set and GSE36133).

Enrichment of KRAS-dependent candidates in mouse models: GSE15326 and GSE32277.

Data of microarray analysis on H2009 transduced with FOSL1 shRNA: GSE76290.

Meta-analysis of PDAC data sets was done as previously described^18^,^63^.

For Kras status predictor on mut KRAS vs WT patients: GSE12667, GSE16515, GSE26566, GSE26939 and Battacharjee *et al*.^65^.

For Kras status predictor on mut KRAS vs mut EGFR: GSE31210, Nguyen *et al*.^68^ and GSE36133.

For Kras status predictor on mut KRAS vs mut EGFR, BRAF or DDR2, EML-ALK4 fusions, or MYC amplification plus no KRAS mutation: https://tcga-data.nci.nih.gov/tcga/tcgaHome2.jsp.

Survival analyses: TCGA LAC, breast invasive carcinoma (BRCA) and lung squamous cell carcinoma (LUSC) RNA-seq data (https://tcgadata.nci.nih.gov/tcga/tcgaHome2.jsp).

Survival in the pancreatic cancer study^24^: https://dcc.icgc.org/repositories/ (under the identifier PACA-AU).

R codes are available upon request.

## Additional information

**How to cite this article:** Vallejo, A. *et al*. An integrative approach unveils FOSL1 as an oncogene vulnerability in KRAS-driven lung and pancreatic cancer. *Nat. Commun.* **8,** 14294 doi: 10.1038/ncomms14294 (2017).

**Publisher's note**: Springer Nature remains neutral with regard to jurisdictional claims in published maps and institutional affiliations.

## Supplementary Material

Supplementary Information Supplementary Figures and Supplementary Tables

Peer Review File
