# Supplementary material for: An integrative approach unveils FOSL1 as an oncogene vulnerability in KRAS-driven lung and pancreatic cancer
Source: Nat Commun. 2017 Feb 21;8:14294. doi: 10.1038/ncomms14294 (PMC5321758; doi:10.1038/ncomms14294)
Supplement: Supplementary Information — Supplementary Figures and Supplementary Tables [file ncomms14294-s1.pdf]

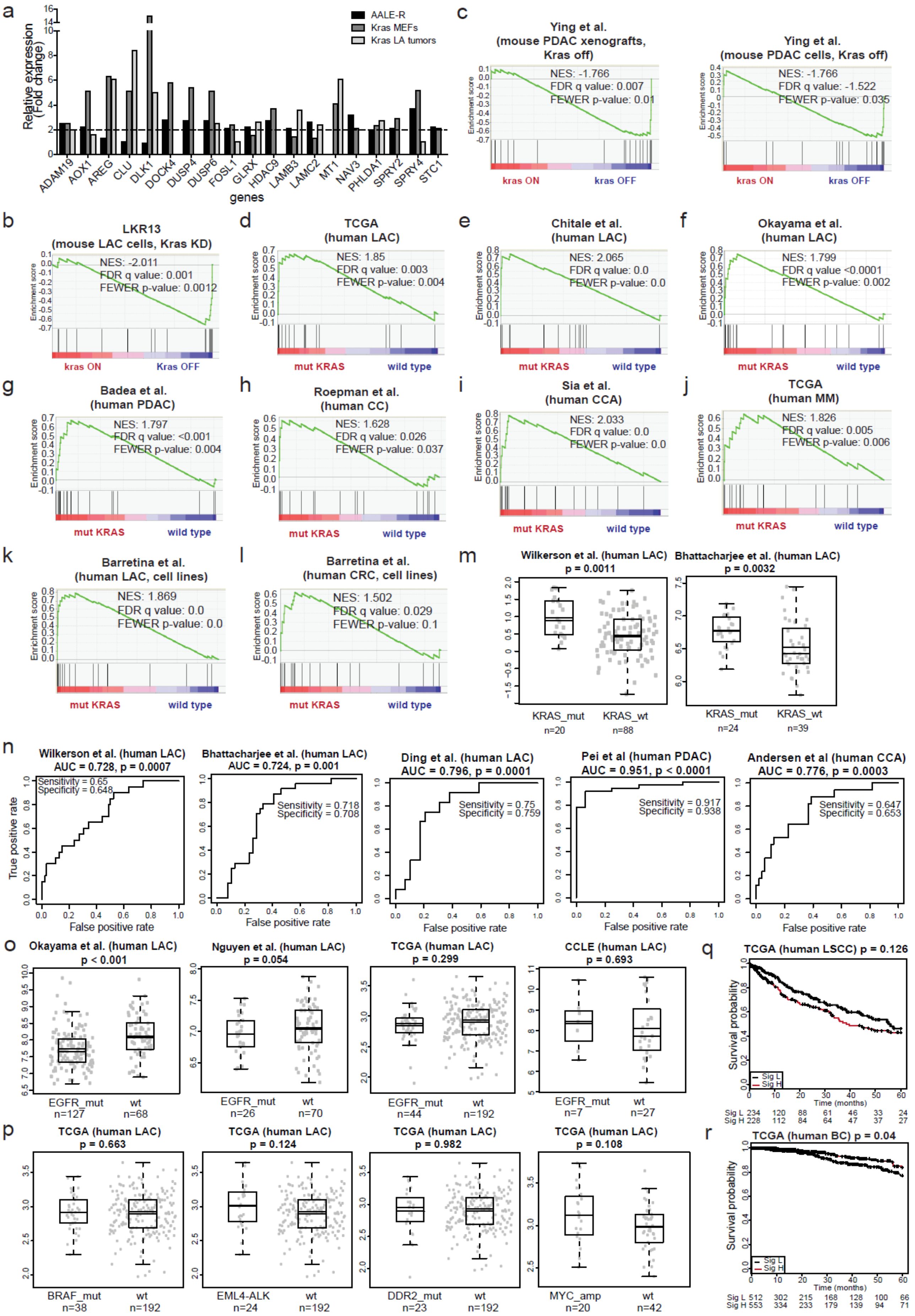
a
Relative expression (Fold change)
AALE-R
Kras MEFs
Kras LA tumors
ADAM19
AOX1
AREG
CLU
DLK1
DOCK4
DUSP4
DUSP6
FOSL1
GLRX
HDAC9
LAMB3
LAMC2
MT1
NAV3
PHLDA1
SPRY2
SPRY4
STC1
genes
c
Ying et al. (mouse PDAC xenografts, Kras off)
NES: -1.766
FDR q value: 0.007
FEWER p-value: 0.01
kras ON
kras OFF
Ying et al. (mouse PDAC cells, Kras off)
NES: -1.766
FDR q value: -1.522
FEWER p-value: 0.035
kras ON
kras OFF
b
LKR13 (mouse LAC cells, Kras KD)
NES: -2.011
FDR q value: 0.001
FEWER p-value: 0.0012
kras ON
Kras OFF
d
TCGA (human LAC)
NES: 1.85
FDR q value: 0.003
FEWER p-value: 0.004
mut KRAS
wild type
e
Chitale et al. (human LAC)
NES: 2.065
FDR q value: 0.0
FEWER p-value: 0.0
mut KRAS
wild type
f
Okayama et al. (human LAC)
NES: 1.799
FDR q value <0.0001
FEWER p-value: 0.002
mut KRAS
wild type
g
Badea et al. (human PDAC)
NES: 1.797
FDR q value: <0.001
FEWER p-value: 0.004
mut KRAS
wild type
h
Roepman et al. (human CC)
NES: 1.628
FDR q value: 0.026
FEWER p-value: 0.037
mut KRAS
wild type
i
Sia et al. (human CCA)
NES: 2.033
FDR q value: 0.0
FEWER p-value: 0.0
mut KRAS
wild type
j
TCGA (human MM)
NES: 1.826
FDR q value: 0.005
FEWER p-value: 0.006
mut KRAS
wild type
k
Barretina et al. (human LAC, cell lines)
NES: 1.869
FDR q value: 0.0
FEWER p-value: 0.0
mut KRAS
wild type
l
Barretina et al. (human CRC, cell lines)
NES: 1.502
FDR q value: 0.029
FEWER p-value: 0.1
mut KRAS
wild type
m
Wilkerson et al. (human LAC) p = 0.0011
KRAS_mut n=20
KRAS_wt n=88
Bhattacharjee et al. (human LAC) p = 0.0032
KRAS_mut n=24
KRAS_wt n=39
n
Wilkerson et al. (human LAC) AUC = 0.728, p = 0.0007
Sensitivity = 0.65
Specificity = 0.648
Bhattacharjee et al. (human LAC) AUC = 0.724, p = 0.001
Sensitivity = 0.718
Specificity = 0.708
Ding et al. (human LAC) AUC = 0.796, p = 0.0001
Sensitivity = 0.75
Specificity = 0.759
Pei et al (human PDAC) AUC = 0.951, p < 0.0001
Sensitivity = 0.917
Specificity = 0.938
Andersen et al (human CCA) AUC = 0.776, p = 0.0003
Sensitivity = 0.647
Specificity = 0.653
True positive rate
False positive rate
o
Okayama et al. (human LAC) p < 0.001
EGFR_mut n=127
wt n=68
Nguyen et al. (human LAC) p = 0.054
EGFR_mut n=26
wt n=70
TCGA (human LAC) p = 0.299
EGFR_mut n=44
wt n=192
CCLE (human LAC) p = 0.693
EGFR_mut n=7
wt n=27
q
TCGA (human LSCC) p = 0.126
Survival probability
Time (months)
Sig L
Sig H
Sig L 234 120 88 61 46 33 24
Sig H 228 112 84 64 47 37 27
p
TCGA (human LAC) p = 0.663
BRAF_mut n=38
wt n=192
TCGA (human LAC) p = 0.124
EML4-ALK n=24
wt n=192
TCGA (human LAC) p = 0.982
DDR2_mut n=23
wt n=192
TCGA (human LAC) p = 0.108
MYC_amp n=20
wt n=42
r
TCGA (human BC) p = 0.04
Survival probability
Time (months)
Sig L
Sig H
Sig L 512 302 215 168 128 100 66
Sig H 553 334 233 179 139 94 71

Supplementary Figure 1. A. Bar graph representing the expression levels of the 19 indicated genes in the microarrays analyses comparing human lung immortalized broncho-epithelial cells (AALE cells) expressing exogenous *KRAS*^{G12D} over wild-type *KRAS*-expressing cell (black), mutant *KRAS* MEFs over wild-type MEFs (dark grey) and *Kras*^{LA2} mouse lung adenocarcinoma tumors over normal lung tissue (light grey). B-L. Gene set enrichment analysis of mouse (B and C) and human (D-L) cancer data sets comparing either active mutant *KRAS* cells to KRAS-inhibited cells (B and C) or mutant *KRAS* patients to wild-type *KRAS* patients (D-L). +, positive; -, negative. M. Box and whiskers plots summarizing the geometric mean of the 8-gene signature in mutant and wild-type *KRAS* LAC data sets. Each dot is a sample. N. Performance of a univariate classification model for predicting mutant vs. wild-type *KRAS* of LAC, PDAC and CCA patients based on gene expression of the 8-gene signature measured by area under receiver operator characteristics (AUC). O. Box and whiskers plots summarizing the geometric mean of the 8-gene signature in mutant and wild-type *EGFR* LAC. P values obtained using Student's *t* test. P. Box and whiskers plots summarizing the geometric mean of the 8-gene signature in mutant *BRAF, EML4-ALK, DDR2* or amplified *MYC* and wild-type LAC. P values obtained using Student's *t* test. Q and R. Kaplan-Meier plot of lung squamous carcinoma (Q) or breast cancer (R) patients (TCGA data set) stratified by the mean expression of the 8-gene cross-tumors signature. P values obtained using log-rank test (Mantel-Cox).

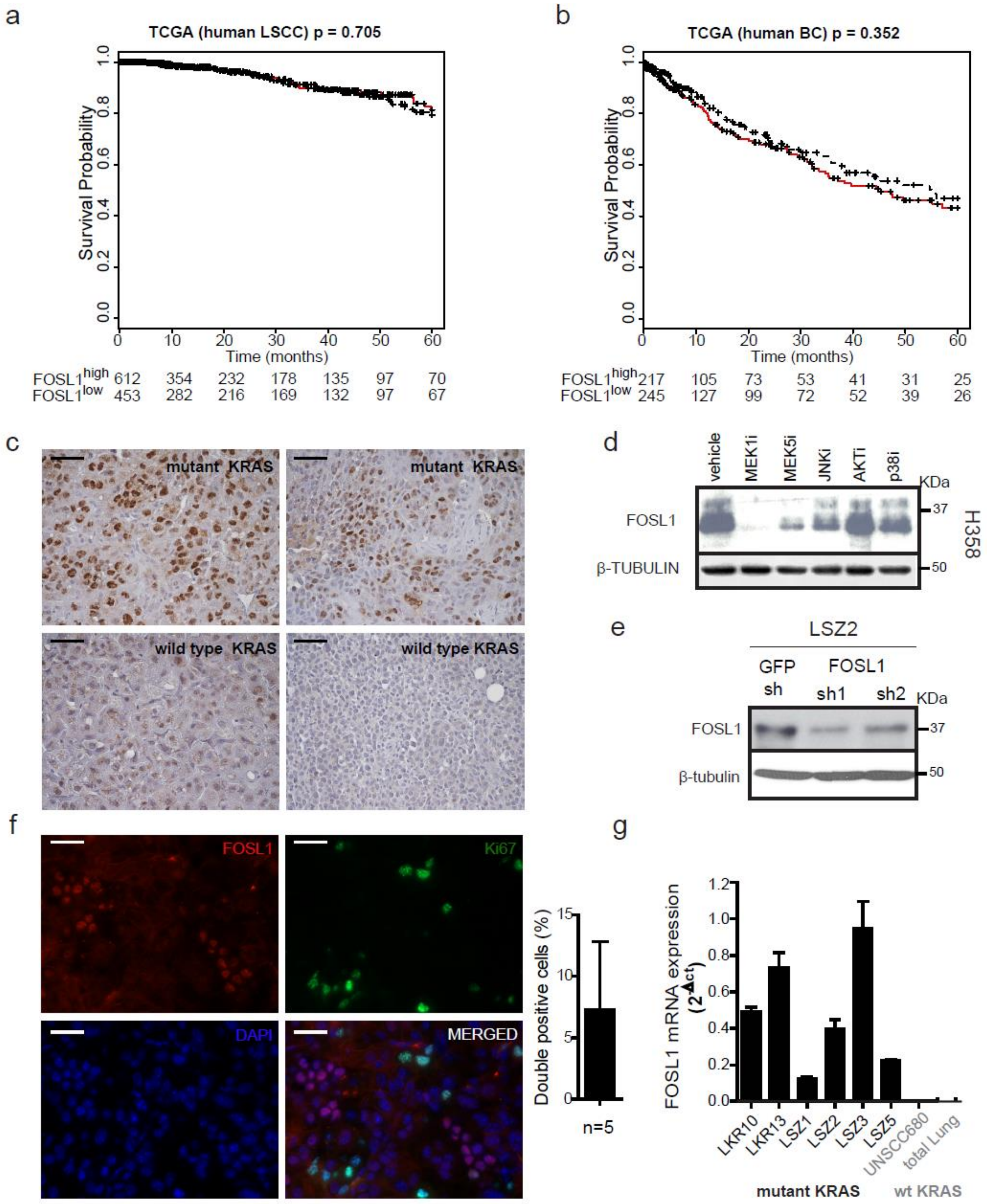

Supplementary Figure 2. A and B. Kaplan-Meier plot of lung squamous carcinoma (A) or breast cancer (B) patients (TCGA data set) stratified by the mean FOSL1 expression. P values obtained using log-rank test (Mantel-Cox). C. Immunohistochemistry of FOSL1 protein in mutant (n=2) and

wild-type (n=2) *KRAS* patient-derived xenografts. D. Western blot of H358 cells treated with U0126 (MEKi, 10 μM), BIX02189 (MEK5i, 10 μM), SB203580 (JNKi, 20 μM), LY294002 (AKTi, 10 μM) and SB203580 (p38i, 20 μM) and probed with indicated antibodies. The experiment was done 3 times. E. Western blot in mouse Kras-driven lung cancer cells for indicated antibodies to show specificity of a FOSL1 antibody in mouse samples. Western blot was done in 3 independent protein lysates for each sample. F. Expression of FOSL1 (red) and Ki67 (green) by immunofluorescence in tumors from $Kras^{LSLG12D}$, $p53^{f/f}$ mice. Scale bar 50 μm. Bar graph indicates percentage of double FOSL1 and Ki67 positive cells in FOSL1-positive tumors. G. qRT-PCR on mutant *Kras* LAC cells, wild-type *Kras* squamous carcinoma cells (UNSCC680) and normal lung. Error bars correspond to s.d. mRNA was obtained from at 3 independent isolates per sample.

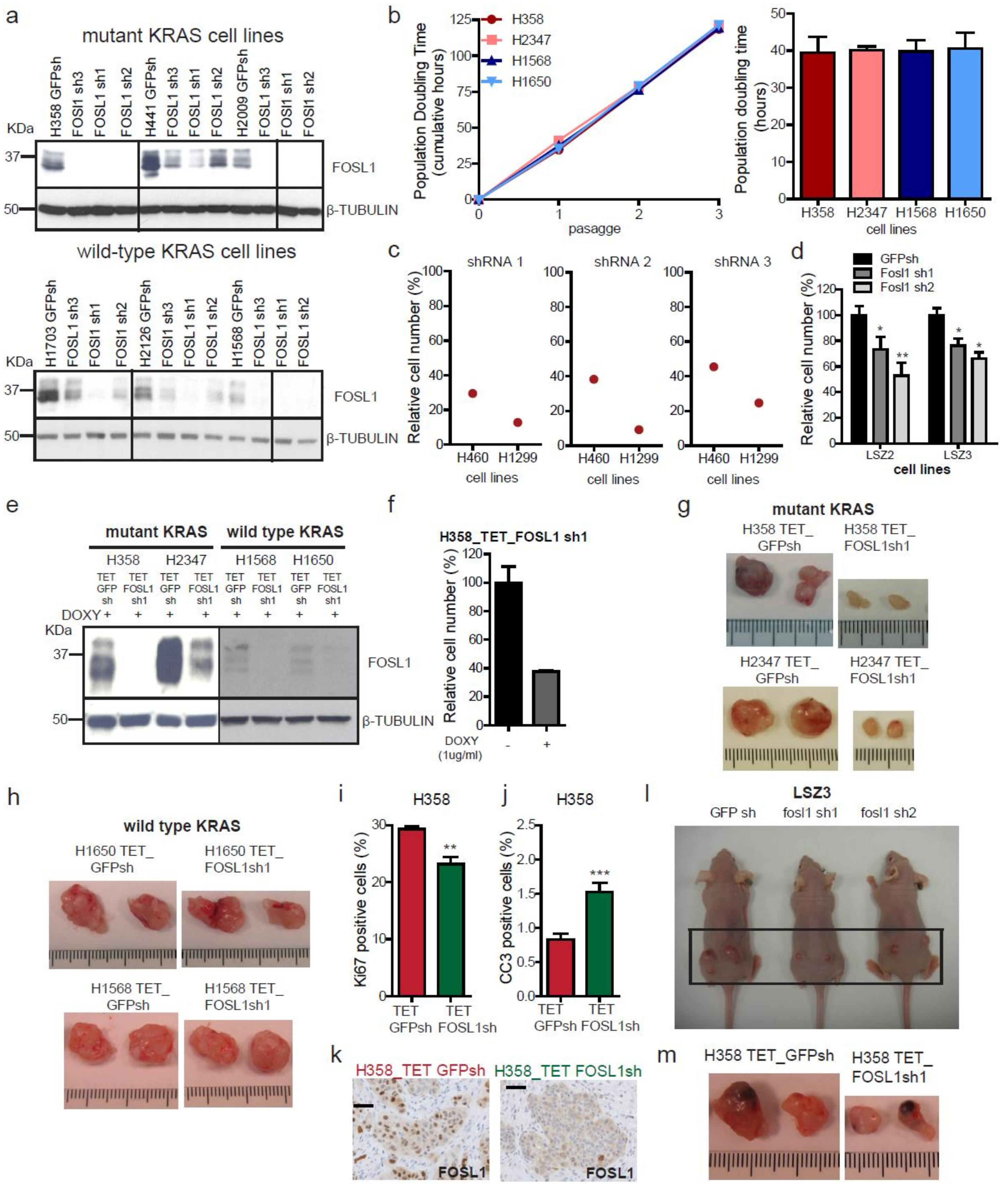

Supplementary Figure 3. A. Western blot for indicated antibodies in mutant and wild-type *KRAS* LAC cell lines after infection with indicated shRNAs. B. Cumulative (left) and average (right) population doubling time of mutant (H358 and H2347) and wild-type (H1568 and H1650) *KRAS* cells.

C. Relative cell number of mutant *RAS*, human large cell carcinoma cell lines assessed by an MTS assay after 3 days in culture. Experiment was performed in triplicate. D. Relative cell number of mutant *Kras*, mouse LAC cell lines (LSZ2 and LSZ3) expressing a control GFP shRNA or two independent shRNAs against *Fosl1* assessed by MTS. Experiment was done 3 times. P values obtained using Student's *t* test. E. Western blot analysis for indicated antibodies in mutant and wild-type KRAS cell lines carrying an inducible FOSL1 shRNA (TET_FOSL1 sh1). Cells were treated with 1 µg/ml doxycycline for 96 h prior to protein collection. Results were similar between 2 different protein isolates. F. Relative cell number of H358 cell line expressing an inducible FOSL1 shRNA. Error bars correspond to s.d. G and H. Representative images of xenografted tumors derived from mutant (H358 and H2347) or wild-type (H1650 and H1568) *KRAS* LAC cells expressing a doxycycline-inducible FOSL1 shRNA or a control GFP shRNA (TET_GFP sh). Error bars correspond to s.e.m. I. Analysis of Ki67 positive cells in representative areas (n=15) from tumors in G (H358). Error bars correspond to s.e.m. P values obtained using Student's *t* test. **, p< 0.01. J. Analysis of cleaved caspase 3 positive cells in representative areas (n=10) of tumors in G. Error bars correspond to s.e.m. P values obtained using Student's *t* test. ***, p< 0.001. K. Immunohistochemistry to detect FOSL1 expression in representative sections of the same tumors as in G. Scale bar 50 µm. *, p<0.05; **, p<0.01; ***, p<0.001. L. Representative images of xenografted tumors derived from mouse mutant *Kras LAC* cells (LSZ3) expressing a *Fosl1* or a control GFP shRNA. M. Representative images of xenografted tumors derived from mutant H358 *KRAS* LAC cells expressing a doxycycline-inducible FOSL1 shRNA or a control GFP shRNA. Doxicicline was administered when tumor volume reached 80 to 100 mm^3.

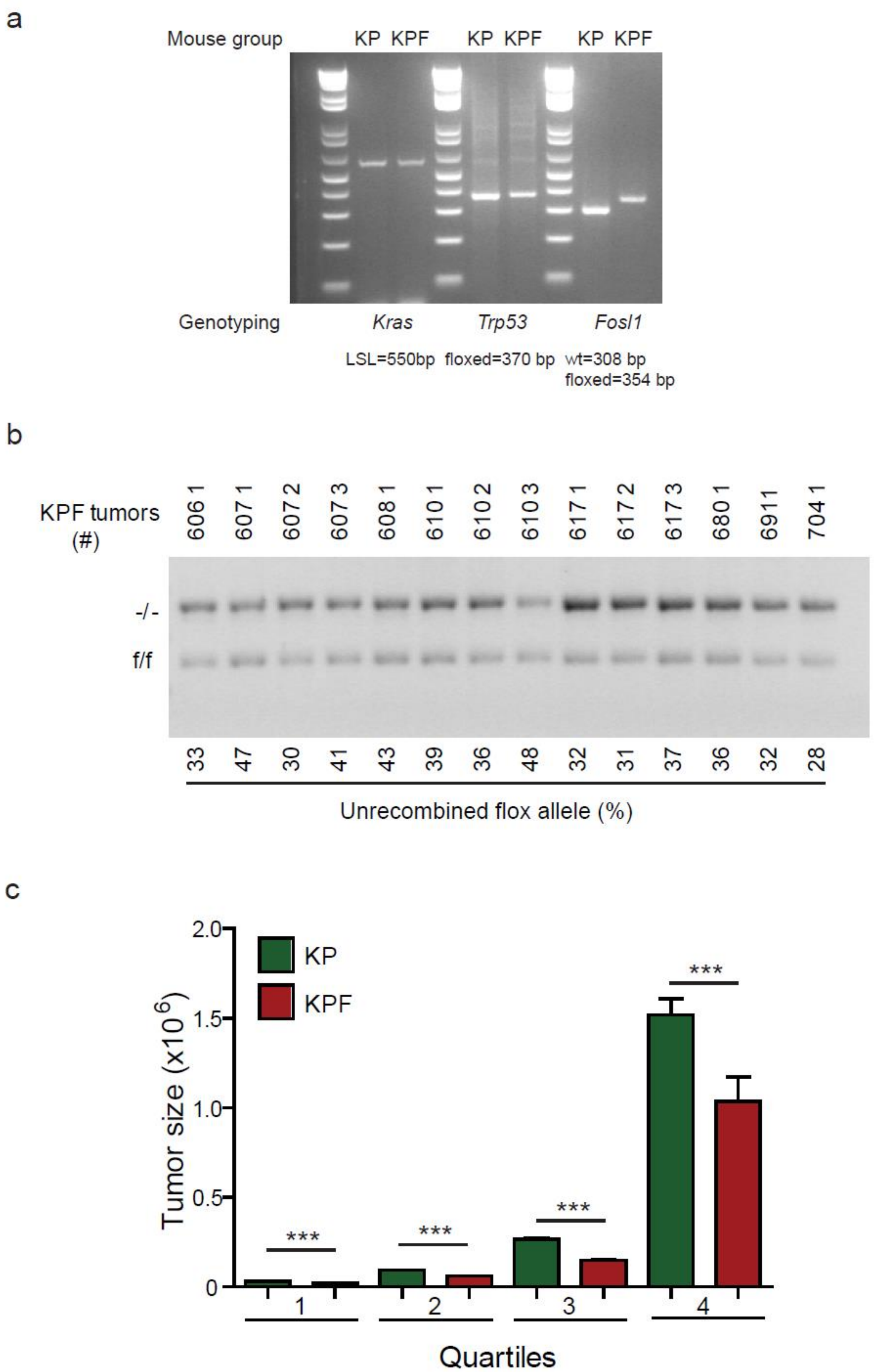

Supplementary Figure 4. A. Genotyping PCR of a mouse from the *Kras*$^{LSL-G12D/+}$; Trp53$^{flox/flox}$; *Fosl1*$^{+/+}$ (KP) and *Kras*$^{LSL-G12D/+}$; *Trp53*$^{flox/flox}$; *Fosl1*$^{flox/flox}$ (KPF) groups. B. PCR to assess the degree of Fosl1 recombined allele in microdissected tumors from KPF mice. C. Tumor size analysis of KP and KPF mice by quartiles.

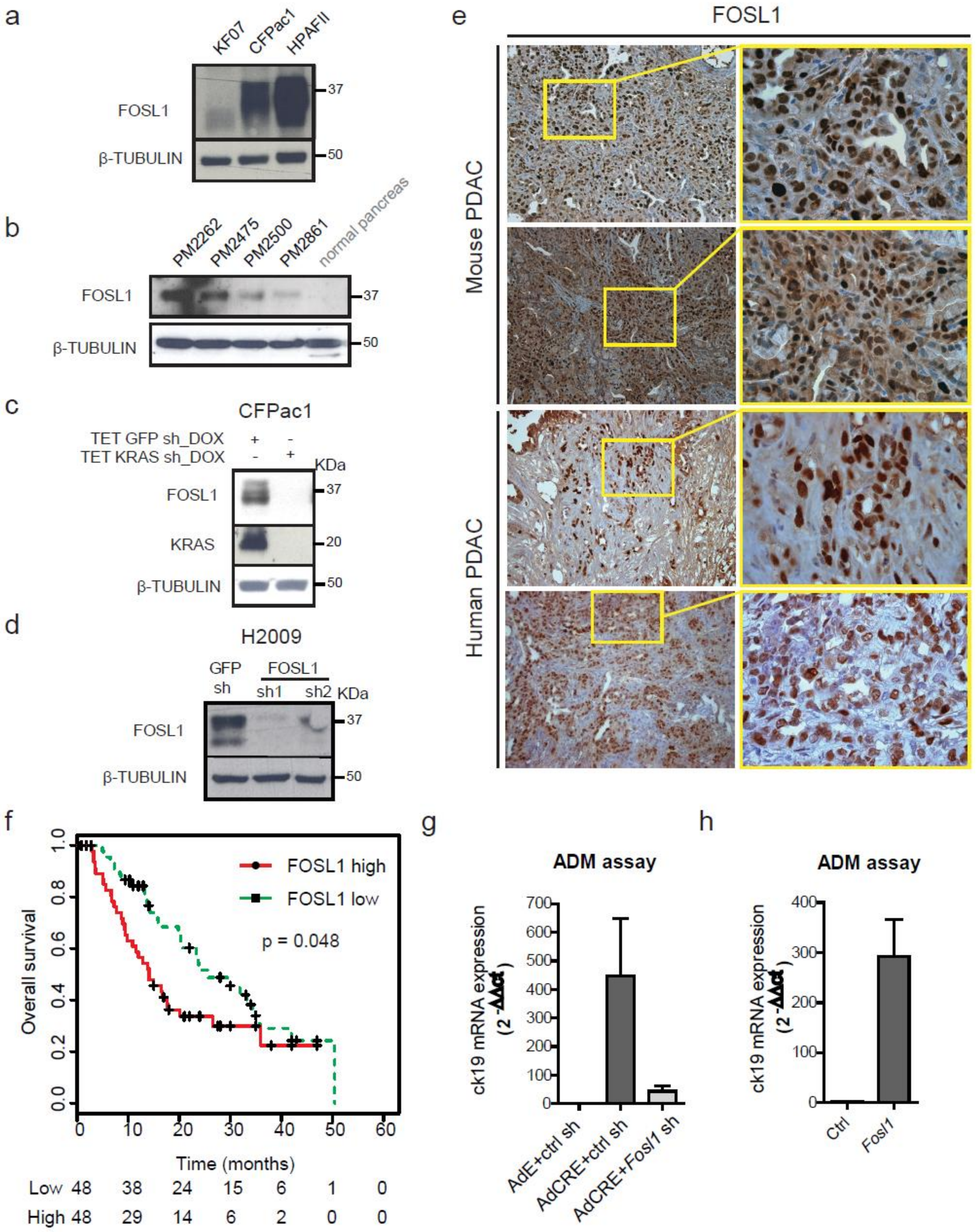

Supplementary Figure 5. A. Western blot for indicated antibodies in immortalized normal epithelial pancreatic cells (KF07) and KRAS-driven PDAC cells (CFPac1 and HPAFII). B. Western blot for indicated proteins in mouse pancreatic cancer cells (black) and normal pancreatic tissue (grey). C. Western blot for indicated antibodies in mutant *KRAS* PDAC cells (CFPac1) carrying a doxyxycline-

inducible KRAS or GFP shRNA. Expression of the shRNA was induced with 1 μg/ml doxycycline for 96 h prior to protein collection. D. Western blot for indicated proteins with a FOSL1 antibody (Santa Cruz Biotech., sc- 376148) specifically used for immunohistochemistry studies to confirm specificity of antibody against human FOSL1 protein. E. Representative images of mouse and human PDAC samples stained with the same FOSL1 antibody as in D. F. Kaplan-Meier plot of PDAC patients for the expression of the 8-gene cross-tumors signature. P values obtained using log-rank test (Mantel-Cox). G. *Ck19* mRNA expression in acinar cells from *Kras*^{LSLG12D} mice treated with control adenovirus (AdE), adenovirus Cre (AdCre) and AdCre plus a shRNA targeting *Fosl1*. H. *Ck19* mRNA expression in acinar cells from *Kras*^{LSLG12D} mice expressing an empty (ctrl) and a *Fosl1*-expressing vector.

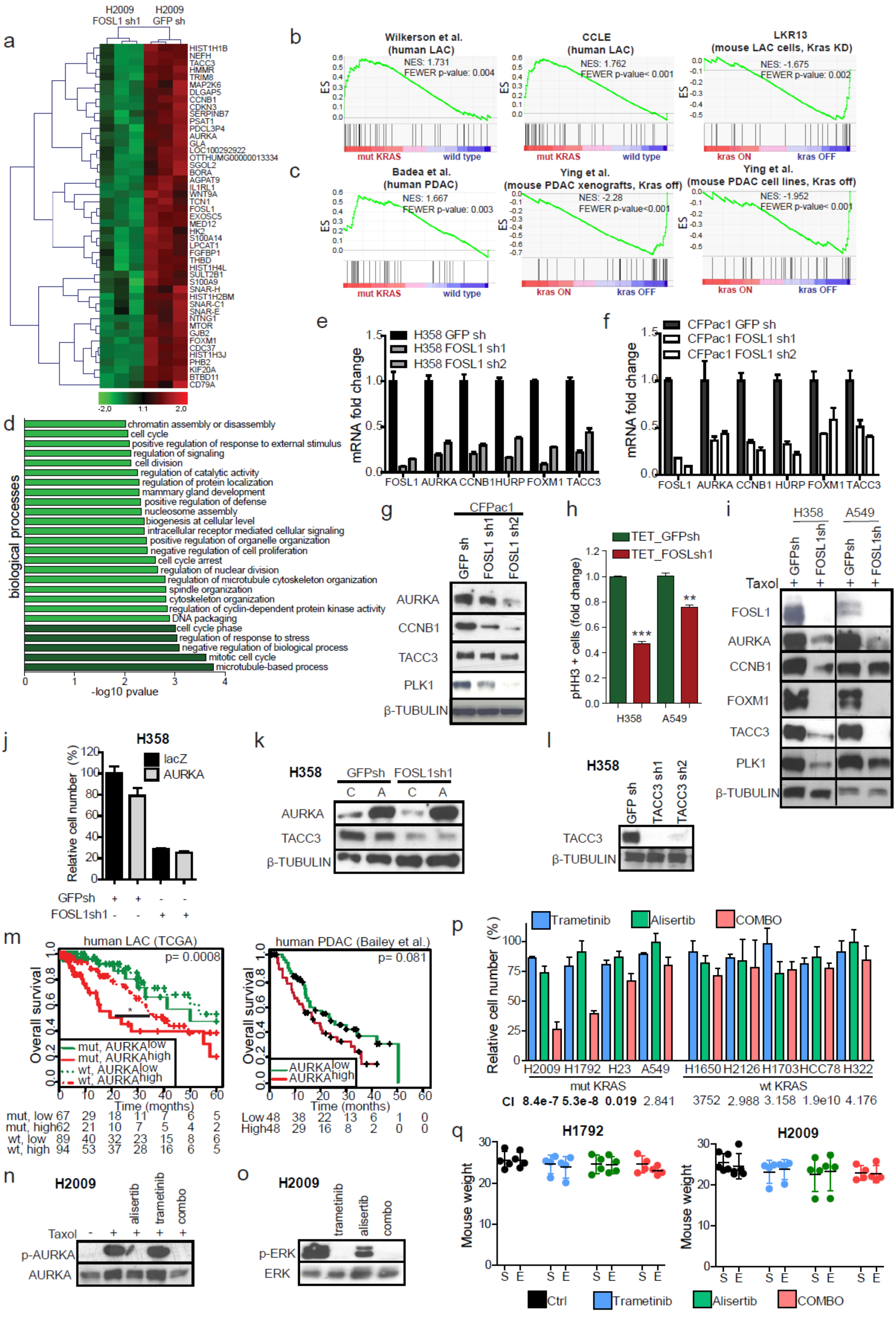

a
H2009 FOSL1 sh1
H2009 GFP sh
HIST1H1B
NEFH
TACC3
HMMR
TRIM8
MAP2K6
DLGAP5
CCNB1
CDKN3
SERPINB7
PSAT1
PDCL3P4
AURKA
GLA
LOC100292922
OTTHUMG00000013334
SGOL2
BORA
AGPAT9
IL1RL1
WNT9A
TCN1
FOSL1
EXOSC5
MED12
HK2
S100A14
LPCAT1
FGFBP1
THBD
HIST1H4L
SULT2B1
S100A9
SNAR-H
HIST1H2BM
SNAR-C1
SNAR-E
NTNG1
MTOR
GJB2
FOXM1
CDC37
HIST1H3J
PHB2
KIF20A
BTBD11
CD79A
-2,0
1:1
2,0
b
Wilkerson et al. (human LAC)
NES: 1.731
FEWER p-value: 0.004
mut KRAS
wild type
CCLE (human LAC)
NES: 1.762
FEWER p-value< 0.001
mut KRAS
wild type
LKR13 (mouse LAC cells, Kras KD)
NES: -1.675
FEWER p-value: 0.002
kras ON
kras OFF
c
Badea et al. (human PDAC)
NES: 1.667
FEWER p-value: 0.003
mut KRAS
wild type
Ying et al. (mouse PDAC xenografts, Kras off)
NES: -2.28
FEWER p-value<0.001
kras ON
kras OFF
Ying et al. (mouse PDAC cell lines, Kras off)
NES: -1.952
FEWER p-value< 0.001
kras ON
kras OFF
ES
d
biological processes
chromatin assembly or disassembly
cell cycle
positive regulation of response to external stimulus
regulation of signaling
cell division
regulation of catalytic activity
regulation of protein localization
mammary gland development
positive regulation of defense
nucleosome assembly
biogenesis at cellular level
intracellular receptor mediated cellular signaling
positive regulation of organelle organization
negative regulation of cell proliferation
cell cycle arrest
regulation of nuclear division
regulation of microtubule cytoskeleton organization
spindle organization
cytoskeleton organization
regulation of cyclin-dependent protein kinase activity
DNA packaging
cell cycle phase
regulation of response to stress
negative regulation of biological process
mitotic cell cycle
microtubule-based process
-log10 pvalue
e
mRNA fold change
H358 GFP sh
H358 FOSL1 sh1
H358 FOSL1 sh2
FOSL1 AURKA CCNB1 HURP FOXM1 TACC3
f
mRNA fold change
CFPac1 GFP sh
CFPac1 FOSL1 sh1
CFPac1 FOSL1 sh2
FOSL1 AURKA CCNB1 HURP FOXM1 TACC3
g
CFPac1
GFP sh
FOSL1 sh1
FOSL1 sh2
AURKA
CCNB1
TACC3
PLK1
β-TUBULIN
h
TET_GFPsh
TET_FOSLsh1
pHH3 + cells (fold change)
***
**
H358
A549
i
H358
A549
Taxol
GFPsh
FOSL1sh
FOSL1
AURKA
CCNB1
FOXM1
TACC3
PLK1
β-TUBULIN
j
H358
lacZ
AURKA
Relative cell number (%)
GFPsh
FOSL1sh1
k
H358
GFPsh
FOSL1sh1
C A C A
AURKA
TACC3
β-TUBULIN
l
H358
GFP sh
TACC3 sh1
TACC3 sh2
TACC3
β-TUBULIN
m
human LAC (TCGA)
p= 0.0008
Overall survival
mut, AURKA low
mut, AURKA high
wt, AURKA low
wt, AURKA high
Time (months)
mut, low 67 29 18 11 7 6 5
mut, high 62 21 10 7 5 4 2
wt, low 89 40 32 23 15 8 6
wt, high 94 53 37 28 16 6 5
human PDAC (Bailey et al.)
p= 0.081
Overall survival
AURKA low
AURKA high
Time (months)
Low 48 38 22 13 6 1 0
High 48 29 16 8 2 0 0
n
H2009
alisertib
trametinib
combo
Taxol
p-AURKA
AURKA
o
H2009
trametinib
alisertib
combo
p-ERK
ERK
p
Trametinib
Alisertib
COMBO
Relative cell number (%)
H2009 H1792 H23 A549
mut KRAS
H1650 H2126 H1703 HCC78 H322
wt KRAS
CI 8.4e-7 5.3e-8 0.019 2.841 3752 2.988 3.158 1.9e10 4.176
q
H1792
H2009
Mouse weight
S E
Ctrl
Trametinib
Alisertib
COMBO

Supplementary Figure 6. A. Heat map of 45 down-regulated genes upon inhibition of FOSL1 in mutant KRAS cells (H2009) by a specific shRNA. B and C. Gene set enrichment analysis of human LAC (B) and PDAC (C) data sets comparing mutant *KRAS* patients to wild-type *KRAS* patients. D. Bar graph representing the Gene Ontology analysis of the biological pathways enriched in genes down-regulated after FOSL1 inhibition. E and F. Q-PCR analysis of mitotic genes in human LAC (E) and PDAC (F) cells transduced with 2 shRNAs to FOSL1.G. Western blot analysis of mitotic genes in CFPac1 PDAC cells after FOSL1 inhibition. H. Cell cycle analysis of pHH3 positive cells in mutant KRAS LAC (H358 and A549) cell lines expressing an inducible GFP or FOSL1 shRNA (1 μg/ml doxycycline) upon treatment with taxol (0.5 μM). I. Western blot analysis for indicated antibodies in mutant KRAS LAC cells (H358 and A549) carrying an inducible FOSL1 or GFP shRNA. Expression of the shRNA was induced with 1 μg/ml doxycycline for 96 h prior to protein collection. J. Cell proliferation assay (MTS) of H358 cells overexpressing lacZ or AURKA and transduced with a shRNA to FOSL1 and a shRNA control (GFP). K. Western blot of indicated proteins in cell lysates from J. C: lacZ; A: AURKA. L. Western blot of indicated proteins in H358 cells transduced two shRNAs targeting TACC3. M. Survival analysis of LAC patients stratified by *KRAS* status and expression of *AURKA, and* PDAC patients stratified by expression of *AURKA*. P values obtained using log-rank test (Mantel-Cox). N. Western blot for phospho (p)-AURKA and AURKA in H2009 cells treated with alisertib (500 nM), trametinib (500 nM) and both for 3 days. Cells were treated with taxol for the last 20 hours of the experiment to induce mitotic arrest and p-AURKA activation. O. Western blot for phospho (p)-ERK and ERK in H2009 cells treated with alisertib (500 nM), trametinib (nM) and their combination. P. MTS analysis of mutant and wild-type *KRAS* cells lines treated with alisertib (1 μM), trametinib (1 μM) or both. CI: combination index. Results are average of 4 different independent treatment experiments performed in triplicate. Q. Average tumor weight of mice injected with H2009 or H1792 cell lines and orally administered vehicle, alisertib (25 mg/kg), trametinib (1 mg/kg) or both. S: start of experiment; E: end of experiment.

Fig. 2b

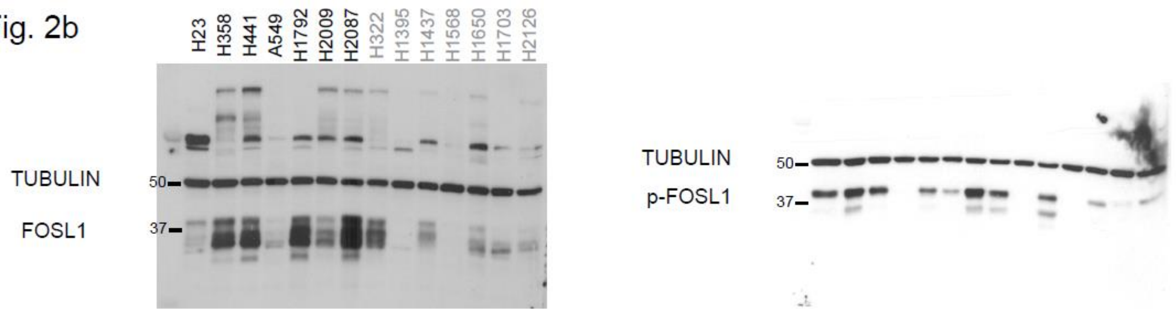
H23
H358
H441
A549
H1792
H2009
H2087
H322
H1395
H1437
H1568
H1650
H1703
H2126
TUBULIN
50
FOSL1
37
TUBULIN
50
p-FOSL1
37

Fig. 2c

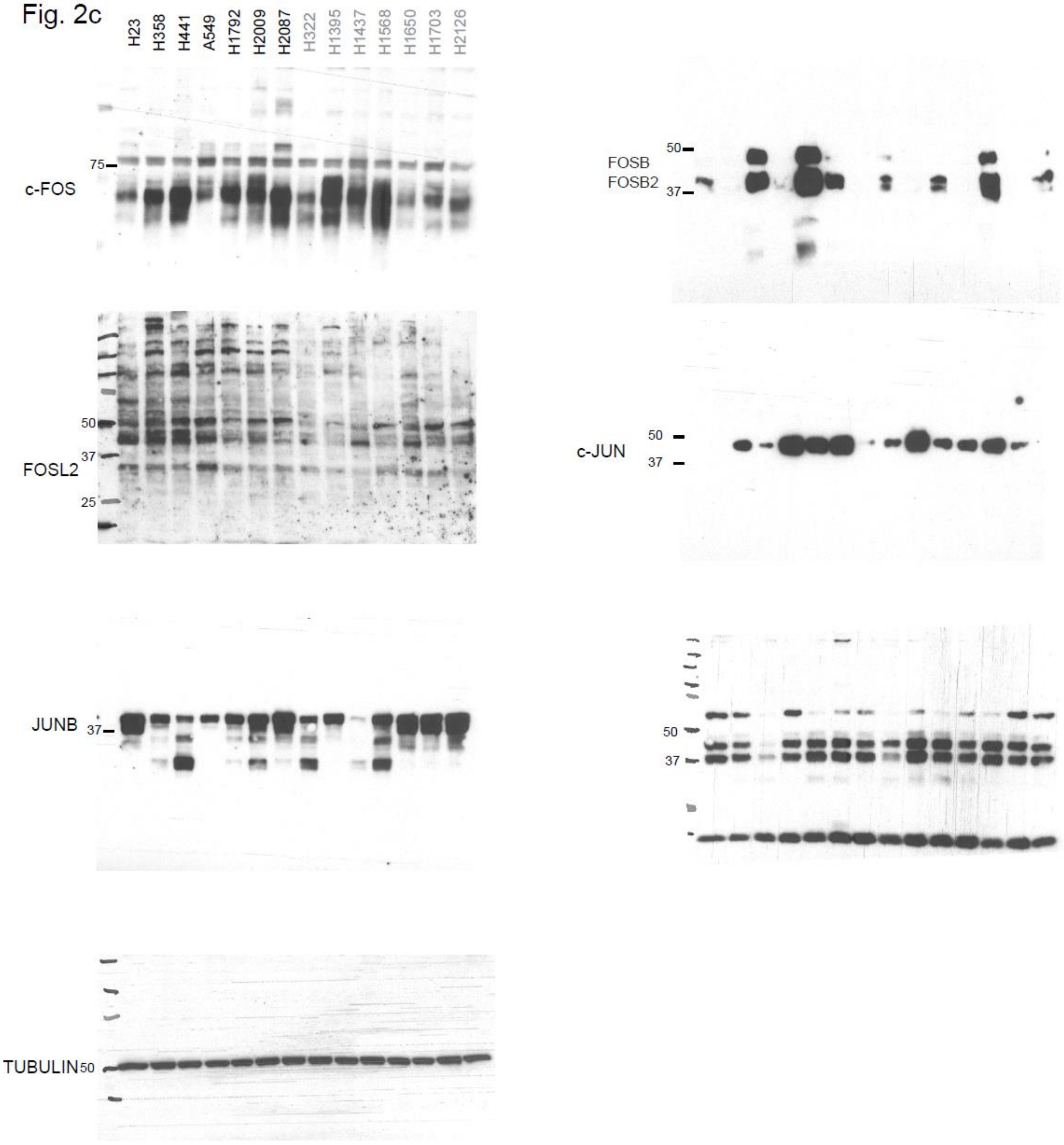
H23
H358
H441
A549
H1792
H2009
H2087
H322
H1395
H1437
H1568
H1650
H1703
H2126
75
c-FOS
50
FOSB
FOSB2
37
50
37
FOSL2
25
50
c-JUN
37
JUNB
37
50
37
TUBULIN
50

Fig. 2d

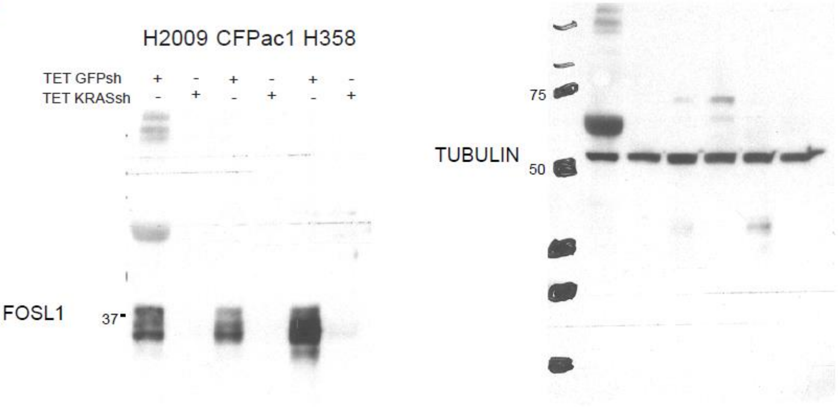

Fig. 2e

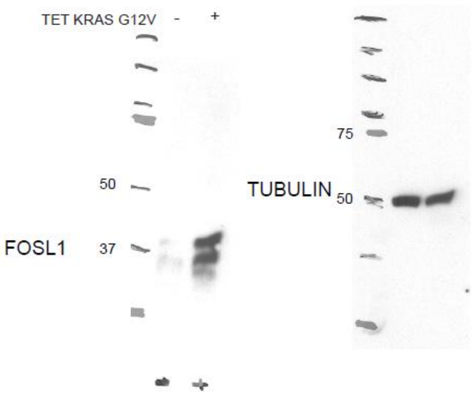

Fig. 2f

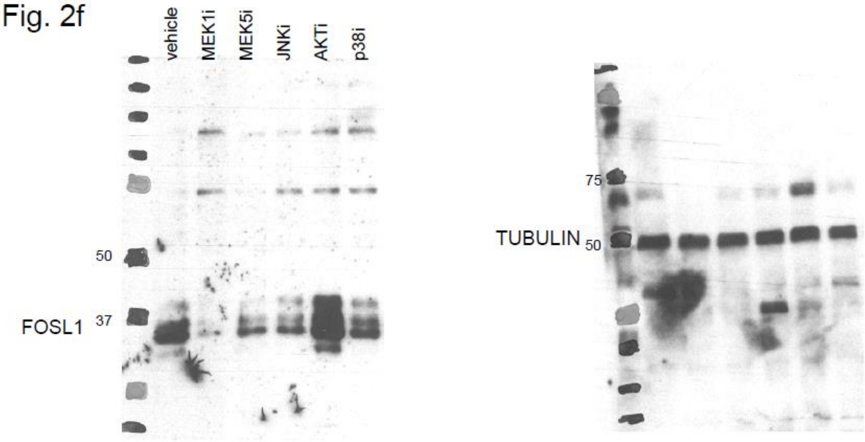

Fig. 2i

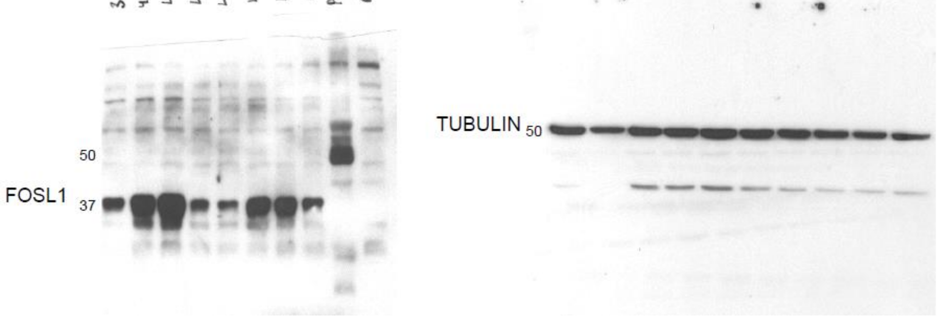

Supplementary Figure 7. Uncropped blots corresponding to Figure 2.

Fig. 5j

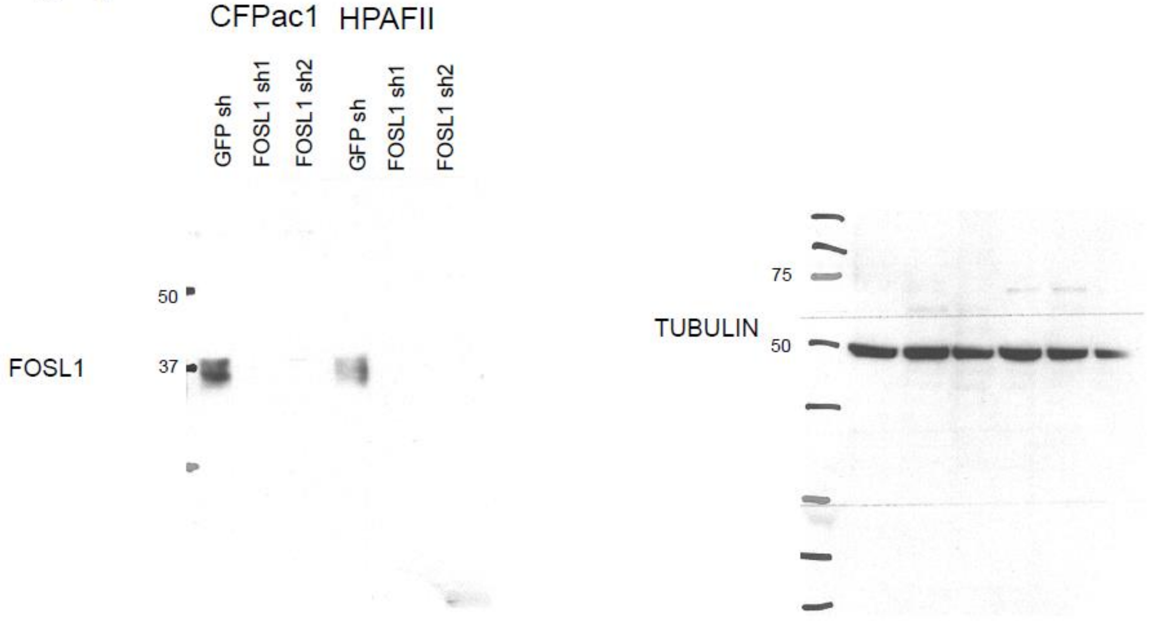

Supplementary Figure 8. Uncropped blots corresponding to Figure 5.

Fig. 6e

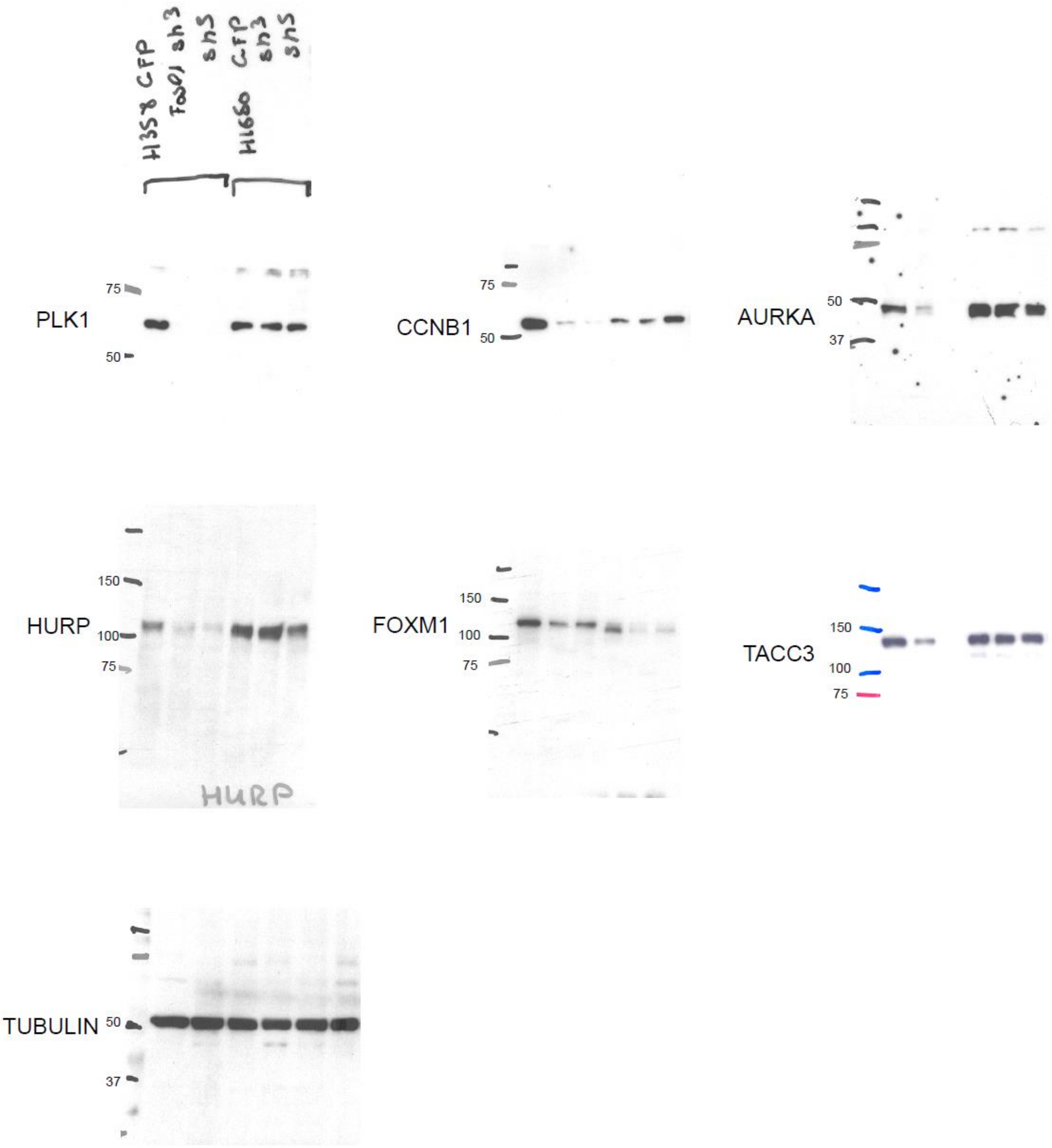

Supplementary Figure 9. Uncropped blots corresponding to Figure 6.

Name	Probeset.x	AALE.MutvsWt.logFC	AALE.MutvsWt.B	Probeset.z	MEFs.MutvsWt.logFC	MEFs.MutvsWt.B	Probeset.d	LA2.MutvsN.logFC	LA2.MutvsN.B
ADAM19	209765_at	1.314214507	1.289626239	1418402_at	1.326806216	-3.223111552	103554_at	1.006791676	19.58707548
AOX1	205083_at	1.111810683	1.789711993	1419435_at	2.339078512	7.349766337	104011_at	0.663907337	2.16102608
AREG	205239_at	0.413419376	-3.888873302	1421134_at	2.655429581	0.165757475	99915_at	2.606357084	35.33778089
CLU	222043_at	0.035780218	-6.595316803	1454849_x_at	2.358785308	0.48596376	95286_at	3.066041629	32.90870974
DLK1	209560_s_at	-0.116535998	-6.201875201	1449939_s_at	3.908639956	9.916748259	101975_at	2.326707281	4.179971992
DOCK4	205003_at	1.505532582	2.935051091	1431114_at	2.545906488	3.622647666	NA	NA	NA
DUSP4	204014_at	1.44105697	3.064072966	1428834_at	2.431045858	0.334895356	NA	NA	NA
DUSP6	208891_at	1.429643604	3.003890749	1415834_at	2.351216618	3.815782947	93285_at	1.300014292	16.25728497
FOSL1	204420_at	1.075107627	2.965207783	1417488_at	1.290424434	0.821459199	99835_at	-0.03760981	-7.458848546
GLRX	209276_s_at	1.110123083	3.165966855	1416592_at	0.620143175	-0.377596367	95722_at	1.358422991	26.07477847
HDAC9	205659_at	1.407177541	5.013290395	1434572_at	1.900903875	0.57901606	NA	NA	NA
LAMB3	209270_at	1.052866705	0.870797535	1417812_a_at	0.53102814	-3.691679671	92759_at	1.854269498	38.85552557
LAMC2	207517_at	1.382477096	1.899102543	1421279_at	0.357314463	-5.382765808	100428_at	1.259546633	42.34499896
NAV3	204823_at	1.681084969	4.322071983	1456144_at	1.093424252	1.432516916	NA	NA	NA
PHLDA1	217997_at	0.976798614	2.237297511	1418835_at	1.218915852	0.914333008	160829_at	1.434815126	17.37798679
SPRY2	204011_at	1.086896567	0.24856866	1436584_at	1.525148674	6.922276652	NA	NA	NA
SPRY4	221489_s_at	1.876731877	5.610182601	1445669_at	2.380211422	6.817248966	98278_at	-0.018891529	-7.671968992
STC1	204597_x_at	1.152724839	1.394438561	1450448_at	1.074527586	0.216654677	NA	NA	NA
MT1	NA	NA	NA	1451612_at	2.051313888	0.388564522	93573_at	2.612437655	54.67021075

a. Mut: mutant

b. Wt: wild type

c. FC: fold change

Supplementary Table 1. Meta-analysis of mouse and human data sets identifies 19 upregulated genes dependent on mutant KRAS expression**.** Summary of microarray analysis comparing human lung immortalized broncho-epithelial cells (AALE cells) expressing exogenous $KRAS^{G12D}$ over wild-type KRAS-expressing cell, mutant KRAS MEFs over wild-type MEFs and $Kras^{LA2}$ mouse lung adenocarcinoma tumors over normal lung tissue. Selected genes include those with a LogFC>1 and B>0 in at least 2 out of the 3 microarray analyses.

GEO	Sample	SitePrimary_lung	HistologySubtype_lung	Histology_AC	KRAS status
GSM886858	A549	lung	non_small_cell_carcinoma	carcinoma	KRAS mut
GSM886861	ABC1	lung	non_small_cell_carcinoma	carcinoma	wt
GSM886905	CAL12T	lung	non_small_cell_carcinoma	NA	wt (BrafG466V)
GSM886915	CALU3	lung	adenocarcinoma	carcinoma	wt
GSM886990	DV90	lung	adenocarcinoma	carcinoma	KRAS mut
GSM887034	HCC1171	lung	non_small_cell_carcinoma	NA	KRAS mut
GSM887036	HCC1195	lung	mixed_adenosquamous_carcinoma	carcinoma	NRAS mut
GSM887050	HCC2279	lung	adenocarcinoma	carcinoma	wt (EGFR mut)
GSM887051	HCC2935	lung	non_small_cell_carcinoma	NA	wt (EGFR)
GSM887053	HCC366	lung	mixed_adenosquamous_carcinoma	carcinoma	wt (DDR2 mut)
GSM887055	HCC4006	lung	adenocarcinoma	carcinoma	wt (EGFR)
GSM887056	HCC44	lung	adenocarcinoma	carcinoma	KRAS mut
GSM887059	HCC78	lung	adenocarcinoma	carcinoma	wt (SLC34A2–ROS1 fusion)
GSM887060	HCC827	lung	adenocarcinoma	carcinoma	wt (EGFR mut)
GSM887093	HS229T	lung	adenocarcinoma	carcinoma	wt
GSM887104	HS618T	lung	adenocarcinoma	carcinoma	wt
GSM887285	LXF289	lung	adenocarcinoma	carcinoma	wt
GSM887339	MORCPR	lung	adenocarcinoma	carcinoma	wt
GSM887358	NCIH1355	lung	adenocarcinoma	carcinoma	KRAS mut
GSM887359	NCIH1373	lung	adenocarcinoma	carcinoma	wt
GSM887361	NCIH1395	lung	adenocarcinoma	carcinoma	wt (Braf)
GSM887362	NCIH1435	lung	non_small_cell_carcinoma	carcinoma	wt
GSM887364	NCIH1437	lung	adenocarcinoma	carcinoma	wt (MEK1)
GSM887366	NCIH1563	lung	adenocarcinoma	carcinoma	wt
GSM887367	NCIH1568	lung	non_small_cell_carcinoma	carcinoma	wt
GSM887368	NCIH1573	lung	adenocarcinoma	carcinoma	KRAS mut
GSM887371	NCIH1623	lung	adenocarcinoma	carcinoma	wt
GSM887372	NCIH1648	lung	adenocarcinoma	carcinoma	wt
GSM887373	NCIH1650	lung	bronchioloalveolar_adenocarcinoma	carcinoma	wt (EGFR mut)
GSM887374	NCIH1651	lung	adenocarcinoma	carcinoma	wt
GSM887375	NCIH1666	lung	bronchioloalveolar_adenocarcinoma	carcinoma	wt (BRAFG466V)
GSM887376	NCIH1693	lung	adenocarcinoma	carcinoma	wt
GSM887378	NCIH1703	lung	adenocarcinoma	carcinoma	wt
GSM887379	NCIH1734	lung	adenocarcinoma	carcinoma	KRAS mut
GSM887380	NCIH1755	lung	adenocarcinoma	carcinoma	wt (BRAFG469A)
GSM887381	NCIH1781	lung	bronchioloalveolar_adenocarcinoma	carcinoma	wt (ERBB2)
GSM887382	NCIH1792	lung	adenocarcinoma	carcinoma	KRAS mut

GSM887383	NCIH1793	lung	non_small_cell_carcinoma	carcinoma	wt
GSM887385	NCIH1838	lung	non_small_cell_carcinoma	carcinoma	wt
GSM887390	NCIH1944	lung	non_small_cell_carcinoma	carcinoma	wt
GSM887393	NCIH1975	lung	non_small_cell_carcinoma	carcinoma	EGFR
GSM887394	NCIH2009	lung	adenocarcinoma	carcinoma	KRAS mut
GSM887395	NCIH2023	lung	adenocarcinoma	carcinoma	wt
GSM887397	NCIH2030	lung	non_small_cell_carcinoma	carcinoma	KRAS mut
GSM887401	NCIH2085	lung	adenocarcinoma	carcinoma	wt
GSM887402	NCIH2087	lung	adenocarcinoma	carcinoma	NRAS mut/Braf mut
GSM887405	NCIH2110	lung	non_small_cell_carcinoma	NA	wt (RST1 mutation)
GSM887407	NCIH2122	lung	adenocarcinoma	carcinoma	KRAS mut
GSM887408	NCIH2126	lung	adenocarcinoma	carcinoma	wt
GSM887415	NCIH2228	lung	adenocarcinoma	carcinoma	wt (ALK fusion)
GSM887418	NCIH2291	lung	adenocarcinoma	carcinoma	wt (EGFR mut)
GSM887419	NCIH2342	lung	adenocarcinoma	carcinoma	wt
GSM887420	NCIH2347	lung	adenocarcinoma	carcinoma	KRAS mut
GSM887421	NCIH23	lung	non_small_cell_carcinoma	carcinoma	KRAS mut
GSM887422	NCIH2405	lung	adenocarcinoma	carcinoma	wt
GSM887423	NCIH2444	lung	non_small_cell_carcinoma	NA	Kras mut
GSM887426	NCIH322	lung	adenocarcinoma	carcinoma	wt (KRAS amplification)
GSM887427	NCIH358	lung	bronchioloalveolar_adenocarcinoma	carcinoma	KRAS mut
GSM887428	NCIH441	lung	adenocarcinoma	carcinoma	KRAS mut
GSM887434	NCIH522	lung	non_small_cell_carcinoma	carcinoma	wt
GSM887439	NCIH650	lung	bronchioloalveolar_adenocarcinoma	carcinoma	wt
GSM887448	NCIH838	lung	non_small_cell_carcinoma	carcinoma	wt (KRAS amplification)
GSM887450	NCIH854	lung	adenocarcinoma	carcinoma	wt
GSM887505	PC14	lung	non_small_cell_carcinoma	carcinoma	wt
GSM887532	RERFLCAD1	lung	adenocarcinoma	carcinoma	KRAS mut
GSM887533	RERFLCAD2	lung	adenocarcinoma	carcinoma	KRAS mut
GSM887535	RERFLCKJ	lung	non_small_cell_carcinoma	carcinoma	wt
GSM887536	RERFLCMS	lung	non_small_cell_carcinoma	carcinoma	wt
GSM886897	C2BBE1	large_intestine	carcinoma	C2BBe1	NA / Wild-type
GSM886924	CCK81	large_intestine	carcinoma	CCK-81	NA / Wild-type
GSM886933	CL11	large_intestine	carcinoma	CL-11	p.Q61H,p.V14I
GSM886934	CL14	large_intestine	carcinoma	CL-14	NA / Wild-type
GSM886935	CL34	large_intestine	carcinoma	CL-34	NA / Wild-type
GSM886936	CL40	large_intestine	carcinoma	CL-40	p.G12D
GSM886940	COLO205	large_intestine	carcinoma	COLO 205	NA / Wild-type
GSM886941	COLO320	large_intestine	carcinoma	COLO-320	NA / Wild-type
GSM886943	COLO678	large_intestine	carcinoma	COLO-678	NA / Wild-type

Supplementary Table 2. Annotation of LAC and CRC cell lines from the Cancer Cell line Encyclopedia.

IHC slide	Patient No.	Sex	Age	PDAC Histology	Localization	G1	G2	G3	pTNM	stage	Survival in mo.
1	10596	M	51	ductal adenocarcinoma	head		G2		pT3N0M0	II	12.00
2	51522	F	78	ductal adenocarcinoma	body, tail		G2		pTxNxM1	IVB	14.07
3	49634	F	57	ductal adenocarcinoma	head			G3	pT1NxM1	IVB	9.00
4	55988	F	76	ductal adenocarcinoma	head		G2		pT3N0M0	II	2.25
5	57120	F	78	ductal adenocarcinoma	head		G2		pT3N0M0	II	6.20
6	57020	F	59	ductal adenocarcinoma	head		G2		pT3N0M0	II	7.80
7	56308	M	68	ductal adenocarcinoma	head			G3	pT3N1aM0	III	0.67
8	55503	M	46	ductal adenocarcinoma	head		G2		pT3N0M0	II	8.13
9	58826	M	68	mucinous cystadenocarcinoma invasive	head		G2		pT3N1bM0	II	11.83
10	57647	M	72	undifferentiated carcinoma	head			G3	pT3N1bM0	III	1.80
11	59871	M	74	ductal adenocarcinoma	head		G2		pT3N0M0	II	10.13
12	62999	M	57	ductal adenocarcinoma	body		G2		pTxNxM1	IVB	1.70
13	60608	M	69	ductal adenocarcinoma	head		G2		pT3N0M0	II	4.10
14	62095	M	73	ductal adenocarcinoma	head			G3	pT3N0M1	II	3.97
15	61365	F	84	ductal adenocarcinoma	head			G3	pT3NxM0	II	1.00
16	12753/07	F	68	ductal adenocarcinoma	head		G2		pTxNxMx	?	6.93
17	11961/07	F	81	ductal adenocarcinoma	body		G2		pTxNxMx	?	9.93
18	1916-40/09	F	64	ductal adenocarcinoma	head	G1			pT3N0M0	II	11.10
19	21056	M	80	ductal adenocarcinoma	head			G3	pT4N1aM0	IVA	2.30
20	264126	F	61	ductal adenocarcinoma	head		G2		pT4N1M1 IVB	IVB	1.60
21	267373	F	71	ductal adenocarcinoma	ogon			G3	pT4N0M0	IVA	0.70
22	272369	M	69	mucinous noncystic carcinoma	body			G3	pT4N0M0	IVA	2.37
23	275118	M	53	mucinous cystadenocarcinoma invasive	body		G2		pT3NxMx	III	60.00
24	283726	M	57	ductal adenocarcinoma	head			G3	pT1N0M0	I	3.10
25	291904	M	65	mucinous noncystic carcinoma	head	G1			pT3N1bM0	III	22.00
26	62888	F	73	ductal adenocarcinoma	head			G3	pT4N1bM1	IVB	0.97
27	3037	M	52	ductal adenocarcinoma	head		G2		pT1N0M0	I	1.97
28	41714	F	60	ductal adenocarcinoma	head		G2		pT4N1bM1	IVB	1.10
29	30250	F	75	ductal adenocarcinoma	head		G2		pT2N0M0	I	60.00
30	13374	F	71	ductal adenocarcinoma	body		G2		pT1N0M0	I	60.00
31	36427	M	66	ductal adenocarcinoma			G2		pT3N0M0	II	6.60
32	33441	M	76	ductal adenocarcinoma	head		G2		pT4NxM1	IVB	3.10
33	31201	M	72	ductal adenocarcinoma	head			G3	pT4NxM1	IVB	1.87
34	25956	M	71	ductal adenocarcinoma	head, body			G3	pT2N0M0	I	16.07
35	4173	M	55	ductal adenocarcinoma	head			G3	pT4N0M1 IVB	IVB	5.77
36	21909	F	54	ductal adenocarcinoma	head, body		G2		pT4N1bM1	IVB	16.00

37	49616	M	61	ductal adenocarcinoma			G2		pT3N0M0	II	18.97
38	43302	M	56	ductal adenocarcinoma	head			G3	pT2N0M0	I	23.00
39	43236	F	61	ductal adenocarcinoma	head		G2		pT4N1aM0	IVA	0.50
40	38753	M	50	ductal adenocarcinoma	head		G2		pT4NxM1	IVB	0.90
41	3782	F	77	ductal adenocarcinoma	head		G2		pT4NxM1	IVB	8.83
42	46912	F	74	ductal adenocarcinoma	head			G3	pT4NxM1	IVB	2.07
43	9304	M	59	mixed:ductal adenoca and squamous ca.	head		G2		pT4N0M0	IVA	20.17
44	24255	M	70	ductal adenocarcinoma	head			G3	pT3N1bM0	III	5.10
45	24048	M	67	ductal adenocarcinoma	head, body			G3	pT4N1bM1	IVB	2.80
46	3072	F	67	ductal adenocarcinoma	head		G2		pTxNxM1	IVB	5.20
47	13470	M	78	ductal adenocarcinoma	head			G3	pTxNxM1	IVB	7.00
48	41895	M	68	ductal adenocarcinoma	head			G3	pT4N1aMx	IVA	2.20
49	15943	F	75	ductal adenocarcinoma	head		G2		pTxNxM1	IVB	16.27
50	23007/09	F	51	ductal adenocarcinoma			G2		pT3N1M0	III	2.17
51	13747	F	71	ductal adenocarcinoma			G2		pT3N0M0	II	0.80
52	299764-5	F	65	mucinous cystadenocarcinoma invasive	head		G2		pT2N0M0	IB	14.07
53	300889-2	F	52	ductal adenocarcinoma	head			G3	pTxNxMx	?	12.03
54	313652	F	72	ductal adenocarcinoma	head		G2		pTxNxMx	?	6.10
55	315472-8	M	48	signet ring carcinoma	head			G3	pTxN1M0	III	60.00
56	320316	F	51	ductal adenocarcinoma	head, body			G3	pT4N1M1	IV	6.13
57	329351	M	44	ductal adenocarcinoma	head		G2		pT4N1M1	IV	5.80
58	331091	M	82	ductal adenocarcinoma	head			G3	pT4N1M1	IV	2.20
59	338141	M	48	mucinous noncystic carcinoma	head		G2		pT3N0M0	IIA	8.10
60	340255-1	F	67	anaplastic carcinoma	body			G3	pT3N0M0	IIA	3.03
61	357321-1	F	67	mucinous noncystic carcinomatrzon			G2		pT3N0M0	IIA	9.50
62	359343-1	M	69	ductal adenocarcinoma	head		G2		pT3N0M0	IIA	5.07
63	385507-3	M	61	ductal adenocarcinoma	head		G2		pT3N0M0	IIA	4.40
64	392798-2	M	80	ductal adenocarcinoma	head		G2		pT3N0M0	IIA	4.20
65	394421	F	53	ductal adenocarcinoma	head		G2		pTxNxM1	IV	15.50
66	398813-5a	M	54	mucinous cystadenocarcinoma invasive	head, body		G2		pT3N1M0	IIB	10.70
67	403590-1	M	83	ductal adenocarcinoma	head			G3	pTxNxM1	IV	1.50
68	404894-1	M	72	ductal adenocarcinoma	head		GX		pTxNxM1	IV	7.03
69	410122-2	F	69	mucinous noncystic carcinoma			G2		pT3N0M0	IIA	12.50
70	419082-4a	F	56	mucinous noncystic carcinoma				G3	pT3N0M0	IIA	4.80
71	298162-6	F	46	ductal adenocarcinoma	head		G2		pT3N0M0	IIA	10.20
72	48187	F	67	ductal adenocarcinoma	głowa		G2		pT2N1bM0	III	13.27

Supplementary Table 3. Information on PDAC patients' clinical data.
