# Supplementary material for: An integrative approach unveils FOSL1 as an oncogene vulnerability in KRAS-driven lung and pancreatic cancer
Source: Nat Commun. 2017 Feb 21;8:14294. doi: 10.1038/ncomms14294 (PMC5321758; doi:10.1038/ncomms14294)
Supplement: Peer Review File [file ncomms14294-s2.pdf]

Editorial Note: this manuscript has been previously reviewed at another journal that is not operating a transparent peer review scheme. This document only contains reviewer comments and rebuttal letters for versions considered at Nature Communications.
